# A flexibility-driven delivery strategy for cationic liposomes to enhance tumor penetration and promote membrane fusion-mediated cellular entry

**DOI:** 10.1016/j.mtbio.2026.103370

**Published:** 2026-06-18

**Authors:** Lifeng Luo, Xiaonan Liu, Yuqing Cai, Yiran Zhang, Yifan Zhao, Zhenqiang Song, Ziwen Meng, Tian Xie, Fenghua Meng, Wenxing Gu

**Affiliations:** a School of Pharmacy, Zhejiang Provincial Key Laboratory of Anti-Cancer Chinese Medicines and Natural Medicines, Engineering Laboratory of Development and Application of Traditional Chinese Medicines, Collaborative Innovation Center of Traditional Chinese Medicines of Zhejiang Province, Hangzhou Normal University, Hangzhou, 311121, PR China; b Biomedical Polymers Laboratory, College of Chemistry, Chemical Engineering and Materials Science, Soochow University, Suzhou, 215123, PR China

**Keywords:** Flexibility, Membrane fusion, Tumor permeation, Cationic liposome, Sodium cholate

## Abstract

Cationic liposomes have emerged as a promising delivery vehicle for chemotherapy. However, their strong electrostatic interaction with tumor vascular endothelium and stroma lead to predominant accumulation at the periphery, thereby restricting deep tumor penetration. Herein, we propose a mechanical programming strategy that modulates the flexibility of cationic liposomes to enhance their tissue permeability, delivery efficiency and antitumor efficacy. By incorporating sodium cholate as a flexibilizer, we engineered a cabazitaxel (CTX)-loaded cationic flexible liposome (CTX@CFL) with enhanced membrane fluidity and deformability. This flexibility-driven redesign shifted the cellular uptake mechanism from endocytosis to membrane fusion, facilitating direct cytosolic delivery and bypassing lysosomal degradation. Consequently, CTX@CFL demonstrated superior transendothelial transport, significantly deeper tumor penetration, and enhanced cytotoxicity compared to conventional cationic rigid liposomes. *In vivo*, CTX@CFL achieved markedly improved tumor accumulation, intratumoral distribution, and potent antitumor efficacy with favorable biocompatibility. This work establishes ‘flexibility modulation’ as a core design strategy that fundamentally overcomes the intrinsic delivery barriers of cationic liposomes. It provides a translatable engineering approach to unlock the full therapeutic potential of cationic liposomal systems in solid tumor therapy.

## Graphical abstract

**Figure:**
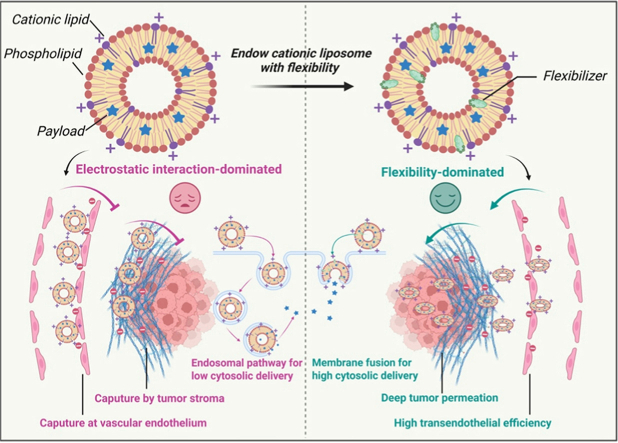

## 1 Introduction

Cationic liposomes constitute promising delivery platforms for chemotherapeutic agents [[1], [2], [3]]. Research into their application for taxane delivery is ongoing, as exemplified by EndoTAG-1®, which is currently in global Phase III clinical trials [4]. Their mechanism involves selective bind to the negatively charged endothelial cells of tumor neovasculature, thereby disrupting angiogenesis and inhibiting tumor growth while sparing healthy blood vessels. A further advantage is their ability to facilitate endosomal escape, improving cytoplasmic drug delivery [[5], [6], [7]]. Despite these strengths, cationic liposomes face significant challenges in the effective delivery of chemotherapeutic payloads [8]. While their positive surface charge promotes rapid interaction and uptake by negatively charged tissues and cells, it simultaneously leads to predominant accumulation at the tumor periphery due to strong electrostatic adsorption. This phenomenon severely limits deep tissue penetration [9]. Within the solid tumor microenvironment, cationic liposomes tend to adhere to the anionic surface of tumor vascular endothelium, which hinders transcytosis and extravasation into the tumor parenchyma. Consequently, the adhesion compromised the effective exploitation of the enhanced permeability and retention (EPR) effect [9,10]. Even when extravasation occurs, cationic liposomes are often rapidly internalized by peripheral cells or sequestered by the negatively charged tumor stroma, restricting their distribution to deeper tumor regions [[11], [12], [13]]. Moreover, although cationic liposomes are recognized for promoting endosomal escape, their efficiency is variable [14,15]. A substantial proportion of the encapsulated drug may still be subjected to lysosomal degradation, resulting in subtherapeutic cytosolic drug concentrations and diminished antitumor efficacy [16]. A series of nanodelivery technologies have been developed to regulate accumulation [[17], [18], [19], [20], [21]], penetration [[22], [23], [24], [25]] and release [[26], [27], [28], [29]] in solid tumors. Overcoming these multifaceted delivery barriers is therefore a critical and unmet need for advancing cationic liposome-based chemotherapy.

Emerging evidence underscores the pivotal role of liposomal elasticity (or mechanical properties) in governing key delivery behaviors, including tumor accumulation, tissue penetration, cellular internalization, and cytosolic drug release [[30], [31], [32], [33], [34]]. Compared to their rigid counterparts, soft nanoparticles with a lower elastic modulus exhibit superior deformability [35]. This property allows them to traverse intercellular pores and physiological constrictions significantly narrower than their nominal diameter [30]. It further facilitates passage through vascular endothelial gaps and the dense interstitial matrix of tumor, which enhances both tumor accumulation and deep tissue penetration [36]. However, highly elastic nanoparticles are also prone to rapid uptake by the reticuloendothelial system (RES), leading to preferential hepatic accumulating and rapid clearance from the systemic circulation [37]. At the cellular level, softer liposomes are more susceptible to internalization *via* membrane fusion, a route that promotes direct cytosolic delivery of the payloads [38]. In contrast, stiffer liposomes are predominantly internalized *via* endocytosis, which routes the encapsulated drug through the endosomal-lysosomal pathway, facing degradation risk [32,39]. Building on this foundation, we hypothesize that modulating the flexibility of cationic liposomes, including fluidity and deformability, represents a promising yet underexplored strategy to overcome delivery limitations and significantly improve tumor-specific targeting and chemotherapy efficacy.

**Scheme 1:**
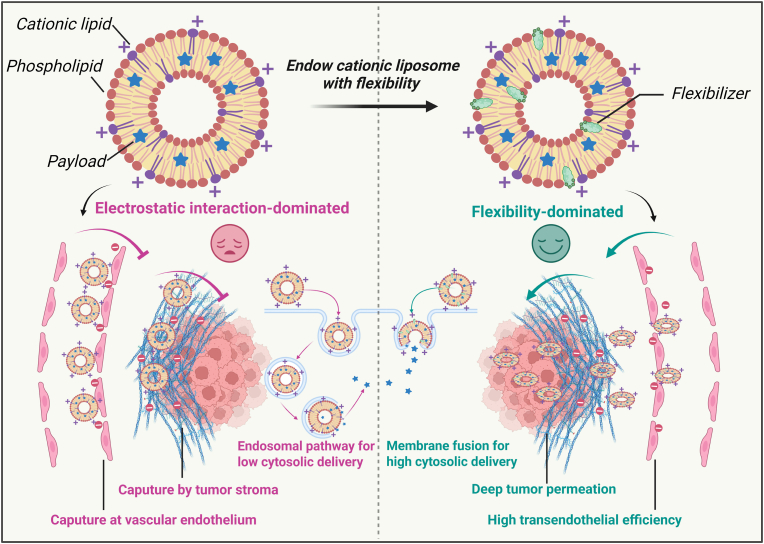
Flexibility-driven the tumor penetration and chemotherapeutic efficacy of cationic liposome in solid tumors.

In this study, we systematically investigated the impact of cationic liposome flexibility on drug delivery dynamics, and explored its modulation as a strategy to enhance tumor-targeted delivery and antitumor efficacy in pancreatic cancer (Scheme 1). Cabazitaxel (CTX) was employed as a model chemotherapeutic agent and encapsulated within a purpose-designed cationic flexible liposome (CTX@CFL). Sodium cholate was identified as the optimal flexibilizer for enhancing the membrane properties of CTX@CFL. We prepared a series of liposomal formulations, including conventional liposome (CTX@L), cationic liposome (CTX@CL), and CTX@CFL with graduated flexibility to comprehensively evaluate the influence of this key parameter on the delivery efficiency and therapeutic outcome of CTX both *in vitro* and *in vivo*. This work not only underscores the potential of cationic flexible liposomes as potent carriers for chemotherapeutics but also proposes a rational design strategy. In this strategy, flexibility modulation serves as a central lever to optimize the tumor delivery performance of cationic liposomal systems in cancer therapy.

## 2 Results and discussion

### 2.1 Screening optimal flexibilizer for cationic liposomes

**Fig. 1:**
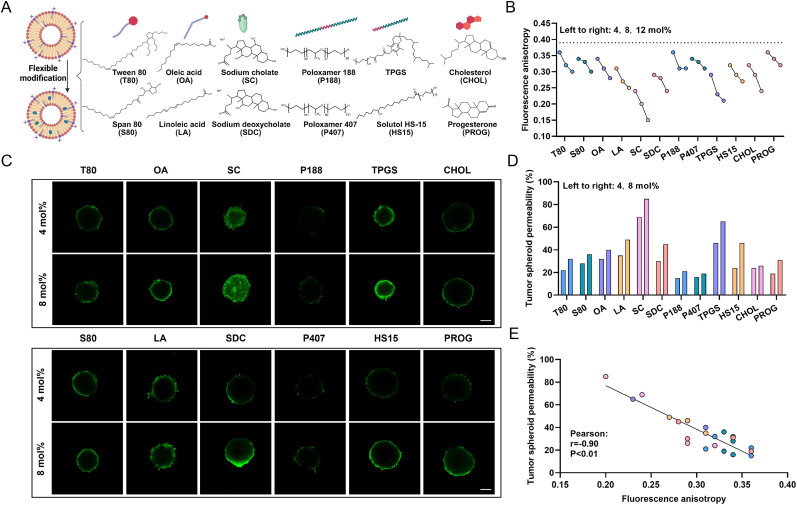
**Screening optimal flexibilizer for cationic liposomes.** (A) Different types of flexibilizers used for the screen of flexible modification of cationic liposomes. (B) Fluorescence anisotropy of DPH-labeled cationic liposomes with different flexibilizers of various concentrations. (C) Fluorescence images of tumor spheroids incubated with coumarin 6-labeled cationic liposomes incorporated with different flexibilizers. Scale bar: 200 μm. (D) Quantified tumor spheroid permeability of cationic liposomes incorporated with different flexibilizers. (E) Correlation analysis of liposomal flexibility (fluorescence anisotropy) and tumor spheroid permeability rate.

Cationic liposome formulations were prepared using the conventional thin-film hydration (Bangham) method, employing 1,2-dioleoyl-3-trimethylammonium-propane (DOTAP) as the cationic lipid. To endow these liposomes with tunable flexibility, we screened a panel of structurally diverse flexibilizers to identify the optimal candidate for constructing cationic flexible liposomes [40,41], typically surfactants and block copolymers including Tween 80, Span 80, oleic acid, linoleic acid, sodium cholate, sodium deoxycholate, poloxamer 188, poloxamer 407, TPGS, Solutol HS-15, cholesterol and progesterone (Fig. 1A). Liposomal membrane fluidity, a key determinant of flexibility, reflecting the rotational mobility of lipid acyl chains within the bilayer at the molecular scale, was assessed by measuring fluorescence anisotropy of incorporated fluorescent probe 1,6-diphenyl-1,3,5-hexatriene (DPH) [42]. Generally, higher membrane fluidity allows greater rotational freedom of DPH molecule, leading to lower anisotropy values. As shown in Fig. 1B, the fluorescence anisotropy of cationic liposomes decreased progressively with increasing molar ratios of the flexibilizers. Notably, sodium cholate-incorporated cationic liposomes exhibited the lowest anisotropy, identifying it as the optimal flexibilizer for modulating cationic liposome properties.

Initial screening revealed that incorporating flexibilizers at 4 and 8 mol% had a negligible impact on particle size and tumor cell viability. In contrast, a 12 mol% incorporation of certain flexibilizers led to reduced particle size and increased cytotoxicity (Fig. S1). Consequently, formulations with 4 and 8 mol% flexibilizers were selected for subsequent tumor permeability evaluation. Permeability was assessed using Coumarin 6-labeled liposomes incubated with 3D tumor spheroid, followed by Z-axis fluorescence confocal imaging. As shown in Fig. 1C, sodium cholate-incorporated cationic liposomes demonstrated the most robust and centralized fluorescence distribution within the tumor spheroid core compared to other flexibilizers-incorporated cationic liposomes. Quantitative analysis of spheroid permeability confirmed the superior performance of the sodium cholate formulation (Fig. 1D). Furthermore, Pearson correlation analysis revealed a strong inverse correlation between fluorescence anisotropy (i.e., lower fluidity) and tumor spheroid permeability rate across these cationic liposomal formulations (Fig. 1E). These results collectively indicate that modulating the flexibility of cationic liposomes is a rational and effective approach to enhancing their tumor-penetrating delivery capability.

### 2.2 Construction and characterization of flexible CTX@CFL

**Fig. 2:**
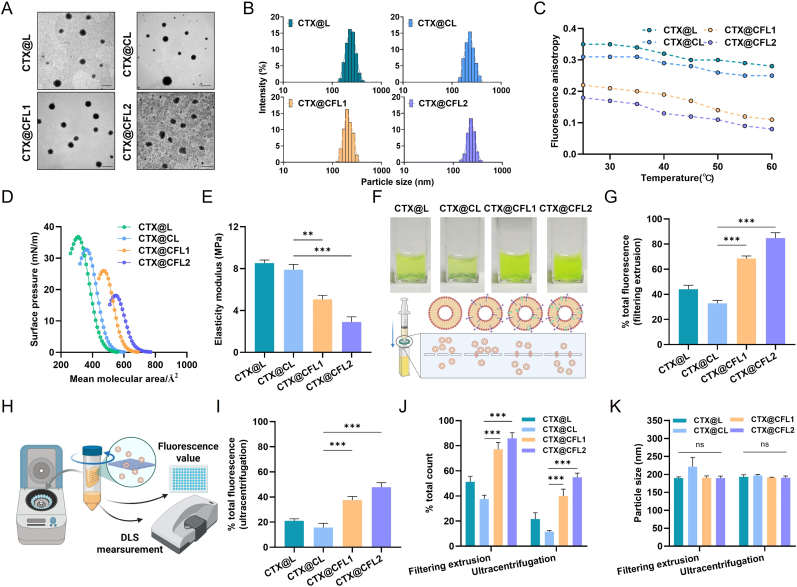
**Flexibility and elasticity of CTX@CFL.** (A) TEM images of different liposome formulations. Scale bar: 200 nm. (B) Particle size and distribution of different liposome formulations. (C) Fluorescence anisotropy of DPH-labeled liposomes from 25°C to 60°C. (D) π-A isotherms of liposomes. (E) Elasticity modulus of liposomes (n = 3). (F) Schematic diagram of filtering extrusion experiment, appearance of coumarin 6-labeled liposomes passed through syringe filters and (G) fluorescence percentage of the filtrate (n = 3). (H) Schematic diagram of ultracentrifugation experiment and (I) fluorescence percentage of the ultrafiltrate (n = 3). (J) The particle count percentage and (K) particle size in filtrate and ultrafiltrate (n = 3). Results are represented as mean ± SD. ns means no significant. ∗∗p < 0.01, ∗∗∗p < 0.001.

Having identified sodium cholate as the optimal flexibilizer, we prepared sodium cholate-incorporated cationic liposomes loaded with CTX (CTX@CFL). Formulations containing 4 and 8 mol% sodium cholate, designated CTX@CFL1 and CTX@CFL2 respectively, were generated to achieve a gradation in liposomal flexibility. Transmission electron microscopy (TEM) revealed that conventional liposome (CTX@L), cationic liposome (CTX@CL) and CTX@CFL1 formulations were spherical and uniform. In contrast, CTX@CFL2, while similar in particle size, exhibited a more irregular morphology, likely attributable to membrane deformation induced by the higher concentration of sodium cholate (Fig. 2A). Compared to CTX@CL, the addition of sodium cholate did not significantly alter the particle size and zeta potential of CTX@CFL1 or CTX@CFL2, which maintained sizes of approximately 180-190 nm and zeta potentials of +23∼+26 mV (Fig. 2B and S2). These liposomal formulations maintained favorable encapsulation efficiency, drug loading capacity and sustained-release behavior *in vitro*, although CTX@CFL2 showed a certain degree of reduction on these indicators (Fig. S3). In addition, all formulations demonstrated good storage stability at 4°C and stability in plasma, but CTX@CFL2 showed more obvious burst release effect (Fig. S4).

The flexibility of liposomes was charactered from three complementary perspectives, including membrane fluidity, elasticity modulus, and deformability. Membrane fluidity, a core component of flexibility, was proved *via* temperature-dependent fluorescence anisotropy measurements using DPH-labeled liposomes (Fig. 2C). As temperature increased, the anisotropy values decreased for all formulations, reflecting the enhanced molecular motion of phospholipid chains during the transition from an ordered gel state to a disordered liquid crystalline state. Crucially, compared to CTX@L and CTX@CL, sodium cholate-containing CTX@CFL maintained significantly lower anisotropy values across entire temperature range, confirming a concentration-dependent enhancement of membrane flexibility.

Elasticity modulus, another important indicator of flexibility, represents the resistance of the lipid monolayer to compression at the mesoscopic scale. A lower elastic modulus indicates higher compressibility and softer mechanical property. Elasticity modulus was quantified using Langmuir-Blodgett (LB) film technology to analyze molecular interactions at the air/water interface [43,44]. Surface pressure versus mean molecular area (π-A) isotherms were recorded during film compression (Fig. 2D). Analysis of key parameters, including the lift-off molecular area (molecule area corresponding to onset of intermolecular interaction) and collapse pressure (maximum surface pressure), revealed that CTX@CFL exhibited larger lift-off areas, earlier collapse points, and lower collapse pressures, indicative of a more disordered phospholipid arrangement and enhanced fluidity (Fig. S5). The compression/elasticity modulus derived from the slope of the linear region in π-A isotherms (Fig. 2E and S5), was markedly lower for CTX@CFL formulations. Specifically, CTX@CFL1 and CTX@CFL2 exhibited 40.63% and 66.02% reductions in elasticity modulus, respectively, relative to CTX@L. This demonstrates that sodium cholate induces a concentration-dependent decrease in decrease rigidity. As an amphiphile, sodium cholate inserts its hydrophobic moiety into phospholipid tails, increasing intermolecular spacing, weakening van der Waals interactions, and thereby facilitating a transition from the liquid condensed phase (LC, where molecules are closely arranged) to the liquid expanded phase (LE, where molecules are loosely arranged), which lower the energy barrier for membrane deformation [45]. DOTAP slightly reduced the elastic modulus of CTX@CL by 7.41% compared to CTX@L, though its effect was substantially weaker than that of sodium cholate. These findings were consistent with the fluidity measurements.

Deformability describes the ability of intact liposomes to undergo reversible shape change when passing through pores narrower than their own diameter, without rupture or content leakage. Deformability of liposomes was assessed using filtering extrusion and ultracentrifugation assays to simulate the shear force of dynamic blood flow and the sustained hydraulic pressure during capillary extravasation, respectively [30,[46], [47], [48]]. In the filtering extrusion experiment, Coumarin 6-labeled liposomes were filtered through 0.2 μm nylon syringe filters (Fig. 2F and G). CTX@CL exhibited the lowest filtrate fluorescence, likely due to electrostatic adsorption to the negatively charged filter membrane. In contrast, CTX@CFL showed significantly higher filtration permeability than CTX@CL, confirming its superior deformability. This trend was corroborated by an ultracentrifugation-based deformation assay using porous ultracentrifugation tube (Fig. 2H). The fluorescence recovery of liposome ultrafiltrate followed the same trend as the results of the filtering extrusion, further supporting the enhanced deformability of CTX@CFL (Fig. 2I). Analysis of derived count rates (DCRs) in the filtrates confirmed a higher percentage of CTX@CFL passed through the barriers compared to CTX@CL (Fig. 2J). Notably, the particle size of CTX@CFL remained unchanged after both procedures, demonstrating that the enhanced passage resulted from reversible deformation rather than structural disintegration (Fig. 2K). In summary, CTX@CFL demonstrated superior penetration through physiologically relevant barriers under both transient shear and sustained pressure while maintaining structural integrity, highlighting its strong, stress-resilient deformability.

### 2.3 Flexibility of CTX@CFL enhanced penetration of intercellular barriers and membrane fusion-mediated tumor cellular internalization

**Fig. 3:**
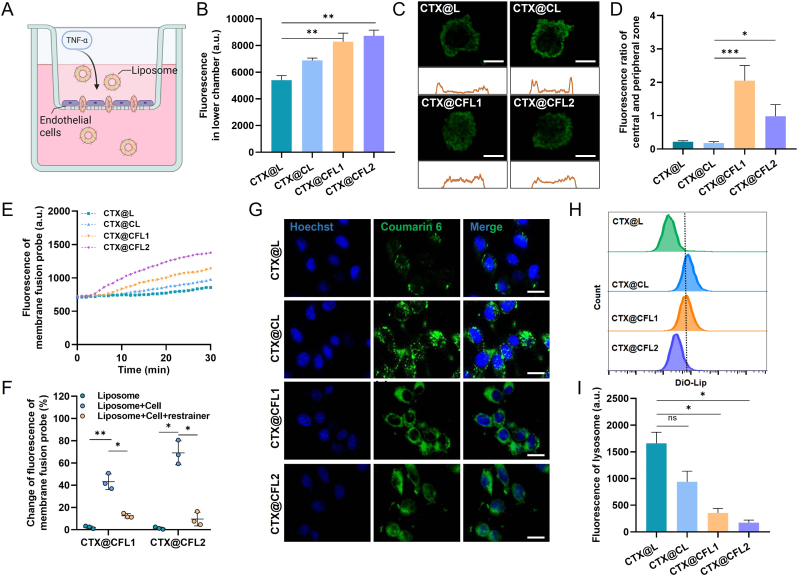
**Flexibility of CTX@CFL enhanced penetration of intercellular barriers and membrane fusion-mediated tumor cellular internalization.** (A) Schematic diagram of transendothelial test of liposomes in a transwell insert system, where endothelial was stimulated with TNF-α to simulate the discontinuous intercellular space of impaired tumor vasculature. (B) Fluorescence intensity in lower chamber of the TNF-α-stimulated transwell system after addition of coumarin 6-labeled liposomes in the upper chamber for 2 h at 37°C. (C) Fluorescence images of Panc02 tumor spheres incubated with coumarin 6-labeled liposomes for 2 h at 37°C. Scale bar: 200 μm. (D) Fluorescence ratio of central and peripheral zone of tumor spheres (n = 3). (E) Fluorescence of membrane fusion probe in liposomes incubated with tumor cells within 30 min. (F) Fluorescence change of membrane fusion probe in liposomes, incubated with tumor cells or pretreated with membrane-fusion restrainer (Z-Phe-Phe-Phe-OH) (n = 3). (G) Fluorescence images of tumor cells incubated with coumarin 6-labeled liposomes at 37°C for 60 min. Scale bar: 20 μm. Quantified fluorescence intensity of (H) tumor cells and (I) their lysosome when cells incubated with coumarin 6-labeled liposomes at 37°C for 60 min (n = 3). Results are represented as mean ± SD. ns means no significant. ∗p < 0.05, ∗∗p < 0.01, ∗∗∗p < 0.001.

Building on the confirmed enhancement of CTX@CFL flexibility, we next evaluated its functional impact on key cellular processes. The transendothelial capability of the liposomes was assessed using a TNF-α to simulated HUVEC monolayer model in a transwell system, which mimics the compromised and hyperpermeable vasculature of tumor (Fig. 3A) [49]. The decreased transepithelial electric resistance (TEER) confirmed the successful construction of this model (Fig. S6A). CTX@CFL exhibited significantly greater transport than CTX@CL, as evidenced by higher fluorescence recovery in the lower chamber (Fig. 3B). This enhancement was specific to the disrupted barrier, as no significant different was observed in a normal endothelial model with intact tight junctions (Fig. S6B). Importantly, liposomes collected from both upper and lower chambers maintained their original sizes, indicating transit without structural disintegration (Fig. S6C). These findings suggest that the high deformability of CTX@CFL enables efficient passage through the enlarged intercellular gaps of diseased tumor vasculature.

Liposome penetration into dense tissue was evaluated using 3D tumor spheroids. Confocal imaging (Fig. 3C) revealed that CTX@L and CTX@CL were predominantly accumulated at the spheroid periphery. In stark contrast, both CTX@CFL1 and CTX@CFL2 penetrated deeply, reaching the central zone of the tumor sphere. Quantitative analysis of the central-to-peripheral fluorescence ratio confirmed that CTX@CFL1 achieved the deepest penetration, indicating superior tumor penetration capacity (Fig. 3D).

Membrane fusion between liposomes and tumor cells was also tested using fluorescent probe Octadecyl Rhodamine B (R18). R18 is self-quenched at high concentrated within liposomal membranes, and fluorescence recovery upon dilution *via* fusion with cell membranes [50]. Thus, the rate and extent of fluorescence recovery directly correlate with membrane fusion efficiency. Incubation with tumor cells, triggered time-dependent fluorescence increases for all R18-labeled liposomes within 30 min (Fig. 3E). CTX@L and CTX@CL showed only modest fluorescence recovery, suggesting limited membrane fusion. In contrast, CTX@CFL exhibited a substantially stronger and more rapid fluorescence increase, reflecting a superior membrane fusion capability conferred by enhanced flexibility. In addition, when pre-added membrane-fusion restrainer (Z-Phe-Phe-Phe-OH), the fluorescence increase of R18 in the group of CTX@CFL1 was obviously inhibited, further indicting the membrane-fusion process (Fig. 3F). Liposomes incorporated with NBD-PE and Rhodamine-DHPE were incubated with tumor cells for the fluorescence resonance energy transfer (FRET) test to detect the membrane fusion [51]. As shown in Fig. S7A, compared with CTX@L and CTX@CL, CTX@CFL showed obviously increased donor NBD fluorescence at different incubation time, which indicated the deceased FRET efficiency and enhanced membrane fusion. To further confirm the membrane fusion pathway, detection of mixing effect the aqueous cavity of liposomes with the cell cytoplasm was performed. Calcein was loaded in liposomes at a self-quenched concentration to detect membrane fusion-mediated delivery of the calcein inside the aqueous cavity of liposomes. As shown in Fig. S7B, the fluorescence of calcein showed poor change when incubated with CTX@L and CTX@CL, but was obviously increased when cells incubated with CTX@CFL, indicating that calcein was released into the cells to relieve its self-quenching. This directly proved that CTX@CFL achieved cellular delivery by membrane fusion. To exclude the membrane-disruptive effect of sodium cholate, DiO-labeled tumor cells were incubated with CTX@CFL for fluorescence imagining (Fig. S8). The tumor cells still showed intact fluorescence signal on cell membrane, confirming that CTX@CFL showed poor influence on cell membrane integrity.

Enhanced membrane fusion suggests a shift away from endocytosis toward cytosolic delivery. To investigate this, we analyzed the cellular uptake of Coumarin 6-labeled liposomes *via* fluorescence microscopy and flow cytometry. Fluorescence images revealed distinct intracellular distribution patterns influenced by liposomal flexibility (Fig. 3G). CTX@L and CTX@CL, with lower flexibility, accumulated mainly in perinuclear regions as bright punctate spots, characteristic of endosomes/lysosomes entrapment following endocytosis. In contrast, cells treated with flexible CTX@CFL displayed a diffuse and homogeneous intracellular fluorescence, consistent with an endocytosis-independent uptake mechanism as previously reported [32,39]. Flow cytometry analysis quantified the total cellular internalization (Fig. 3H). CTX@CL showed higher uptake than CTX@L, attributable to favorable electrostatic interactions with the negatively charged cell membrane. While CTX@CFL1 showed uptake comparable to CTX@CL, whereas CTX@CFL2 exhibited a reduction, possibly due to decreased colloidal stability at highest flexibilizer concentration.

To delineate the primary internalization pathway, cells were pretreated with different inhibitors, including clathrin inhibitor chlorpromazine (CPZ), caveolae inhibitor methyl-β-cyclodextrin (HP-β-CD), macropinocytosis inhibitor amiloride [52], energy deplete agent sodium azide (NaN_3_) and membrane fusion inhibitor Z-Phe-Phe-Phe-OH [53]. As shown in Fig. S9, cellular uptake of CTX@CFL was not obviously affected by CPZ, HP-β-CD, amiloride and NaN_3_, confirming that endocytosis was not its primary internalization route. While, its cellular uptake level was significantly reduced when pretreated with Z-Phe-Phe-Phe-OH, which indicated that CTX@CFL was mainly internalized by tumor cells *via* membrane fusion pathway. We further quantified liposome trafficking to lysosomes by analyzing fluorescence in isolated lysosomal suspension. CTX@CL showed lower lysosomal fluorescence than CTX@L, consistent with partial endosomal escape capability of cationic liposomes. Strikingly, CTX@CFL exhibited a further significantly reduced lysosomal fluorescence compared to CTX@CL. This minimal lysosomal delivery was unaffected by pretreatment with the lysosomal inhibitor bafilomycin A1, conclusively indicating bypass of the endolysosomal pathway (Fig. 3I and S10). Collectively, these results indicate that augmenting cationic liposome flexibility promotes membrane fusion as the dominant uptake mechanism, facilitating direct cytosolic delivery and minimizing endolysosomal sequestration.

In summary, the strategic modulation of cationic liposome flexibility *via* sodium cholate incorporation fundamentally altered its cellular interaction paradigm. This shift from endocytosis to membrane fusion coupled with enhanced deformability, collectively underpinned the superior ability of CTX@CFL to cross cellular and tissue barriers, resulting in markedly improved transendothelial transport and deep tumor penetration *in vitro*.

### 2.4 Flexibility of CTX@CFL improved cytotoxicity toward tumor cells *in vitro*

**Fig. 4:**
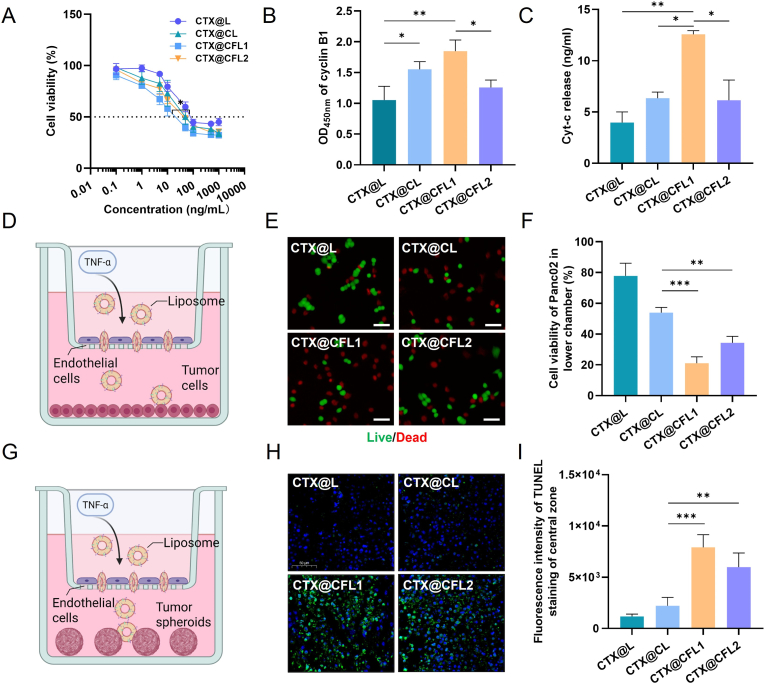
**CTX@CFL improved cytotoxicity toward tumor cells *in vitro*.** (A) Cytotoxicity analysis of liposomes to Panc02 tumor cells (n = 3). (B) Expression level of cyclin B1 and (C) release level of cytochrome C of tumor cells incubated with liposomes for 48 h at 37°C (n = 3). (D) Schematic diagram of tumor cell-killing test of liposomes in a transwell insert system, where endothelial cells and Panc02 tumor cells were seeded at the upper and lower chamber respectively. (E) Live/dead staining and (F) cell viability of tumor cells in the lower chamber of the transwell system (n = 3). Scale bar: 50 μm. (G) Schematic diagram of tumor cell-killing test of liposomes in a transwell insert system, where endothelial cells and Panc02 tumor spheroids were seeded at the upper and lower chamber respectively. (H) Fluorescence images and (I) quantified fluorescence intensity of TUNEL staining tumor sphere. Scale bar: 50 μm. Results are represented as mean ± SD. ∗p < 0.05, ∗∗p < 0.01, ∗∗∗p < 0.001.

Considering the demonstrated improvements in cytosolic delivery, transendothelial transport and tumor sphere penetration achieved by modulating liposome flexibility, we next evaluated the *in vitro* antitumor efficacy of the optimized formulation, CTX@CFL. Consistent with its enhanced cytosolic delivery mechanism, CTX@CFL resulted in significantly stronger cytotoxicity compared to CTX@CL, as reflected by a lower IC_50_ value (Fig. 4A). Notably, CTX@CFL1 exhibited greater cytotoxicity than CTX@CFL2, which correlated with their differential cellular uptake levels. The empty liposomal carriers showed negligible cytotoxicity, confirming that the antitumor effects were attributable to the efficient internalization and altered delivery mechanism of the payloads (Fig. S11). As a microtubule inhibitor, CTX is known to induce G2/M phase cell cycle arrest, upregulate cyclin B1 expression, and promote mitochondrial *via* apoptosis modulation of Bcl-2 family proteins, cytochrome C release, and caspase activation [[54], [55], [56], [57], [58]]. Consistent with its superior cytotoxicity, CTX@CFL1 treatment resulted in the most pronounced upregulation of cyclin B1 and the highest level of cytochrome C release, confirming that the cationic flexible liposome formulation optimally enhances the dual cell-cycle arrest and apoptosis actions of CTX (Fig. 4B and C).

To determine whether superior transendothelial capability of the liposomes translates to enhanced tumor cell killing across a biological barrier, we established a transwell co-culture model. In this system, endothelial cells were seeded in the upper chamber, with Panc02 tumor cells cultured in the lower chamber (Fig. 4D). Following application of liposomal formulations to the upper compartment, tumor cell viability in the lower chamber was measured. Owing to its high transendothelial efficiency, CTX@CFL1 induced the most pronounced cytotoxicity after crossing the endothelial barrier, as demonstrated by live/dead staining and cell viability assays (Fig. 4E and F). Furthermore, to link deep tumor penetration with therapeutic effect in a tissue-like context, we performed TUNEL staining on 3D tumor spheroid sections in the transwell model (Fig. 4G). CTX@CFL1 treatment elicited the strongest apoptotic response specifically within the core region of spheroids, directly aligning with its superior tumor penetration profile (Fig. 4H and I). In summary, a clear positive correlation was established between the delivery performance of the liposomal formulations (governed by flexibility) and their ultimate antitumor efficacy. These findings underscore that modulating liposomal flexibility is a critical strategy to enhance the therapeutic index of chemotherapy by ensuring efficient drug delivery to its intracellular and intratumoral sites of action.

### 2.5 Flexibility of CTX@CFL enhanced tumor accumulation and penetration *in vivo*

**Fig. 5:**
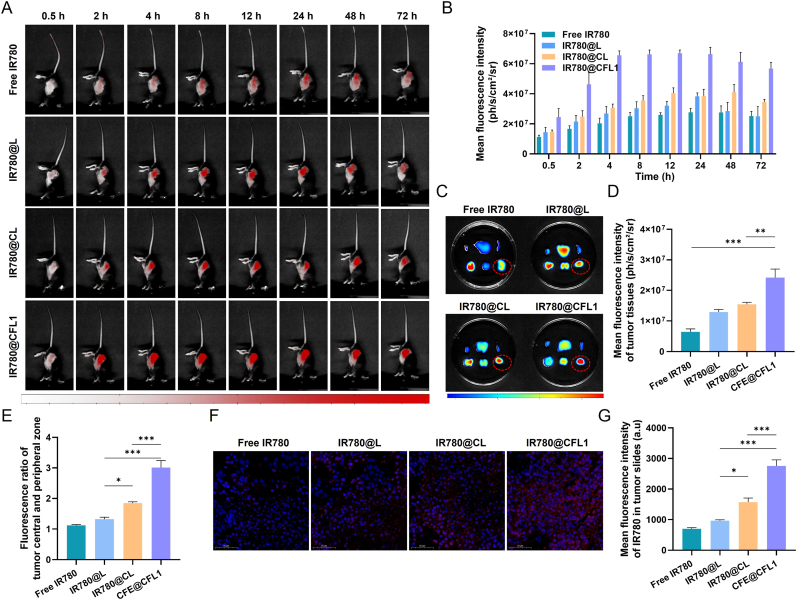
**Flexibility of CTX@CFL enhanced tumor accumulation and penetration *in vivo*.** (A) In vivo fluorescence imaging and (B) quantified mean fluorescence intensity of tumor tissue on Panc02-bearing mice at 72 h after intravenous injection of IR780-labeled liposomes (n = 4). (C) *Ex vivo* fluorescence imaging and (D) quantified mean fluorescence intensity of tumor tissues from Panc02-bearing mice at 72 h after intravenous injection of IR780-labeled liposomes (n = 4). (E) Fluorescence ratio of central and peripheral zone of isolated tumor tissues (n = 4). (F) Fluorescence images and (G) quantified mean fluorescence intensity of IR780 of tumor tissues slice from mice treated with IR780-labeled liposomes (n = 3). Scale bar: 50 μm. Results are represented as mean ± SD. ∗p < 0.05, ∗∗p < 0.01, ∗∗∗p < 0.001.

To confirm the improved *in vivo* tumor targeting and intratumoral distribution in solid tumors, we quantitatively assessed in a subcutaneous pancreatic tumor-bearing mouse model [59,60]. Mice were intravenously administered IR780-labeled liposomal formulations and subjected to real-time fluorescence imaging over 72 h [61]. As illustrated in Fig. 5A and B, tumor-associated fluorescence signals increased in a time-dependent manner across all groups, demonstrating progressive accumulation of liposomal carriers. Throughout the imaging period, the CTX@CFL1 group consistently displayed the most intense fluorescence signals, significantly outperforming CTX@CL and other controls. This sustained high signal intensity indicates superior tumor-targeting affinity and prolonged retention of the flexible cationic liposome formulation. Complementary *ex vivo* imaging of excised tumors and major organs at the 72-h endpoint further validated these findings (Fig. 5C and D). Quantitative analysis revealed that tumor tissues from the CTX@CFL1 group exhibited the highest mean fluorescence intensity, providing direct evidence of its significantly enhanced tumor accumulation. Considering the similar particle size and zeta potential between CTX@CFL1 and CTX@CL1, the improved tumor targeting was mainly attributed to the increased flexibility of the liposomes, rather than the positive charge-mediated interactions and the EPR effect.

We next evaluated the critical parameter of deep tumor penetration by analyzing the spatial distribution of fluorescence within tumor tissues. The fluorescence intensity ratio between central and peripheral tumor zones was calculated as a key metric for penetration depth assessment. CTX@CFL1 achieved the highest center-to-periphery fluorescence ratio (Fig. 5E), indicating a superior ability to reach the tumor core rather than being confined to the periphery. Subsequent histological analysis of tumor sections provided visual confirmation of this phenomenon, with IR780-labeled CTX@CFL1 showing the most pronounced fluorescence signals in the deep, core regions (Fig. 5F and G). Collectively, these *in vivo* imaging analyses provide compelling evidence that the engineering structural flexibility into CTX@CFL1 significantly enhances both tumor accumulation and, crucially, deep tissue penetration. This superior performance, which markedly exceeds that of conventional cationic liposomes (CTX@CL), can be attributed to the flexibility-optimized physicochemical properties that facilitate improved transport across biological barriers, leading to a more homogeneous intratumoral distribution. This addresses a fundamental delivery challenge in solid tumor chemotherapy.

### 2.6 Flexibility of CTX@CFL improved *in vivo* antitumor effect

**Fig. 6:**
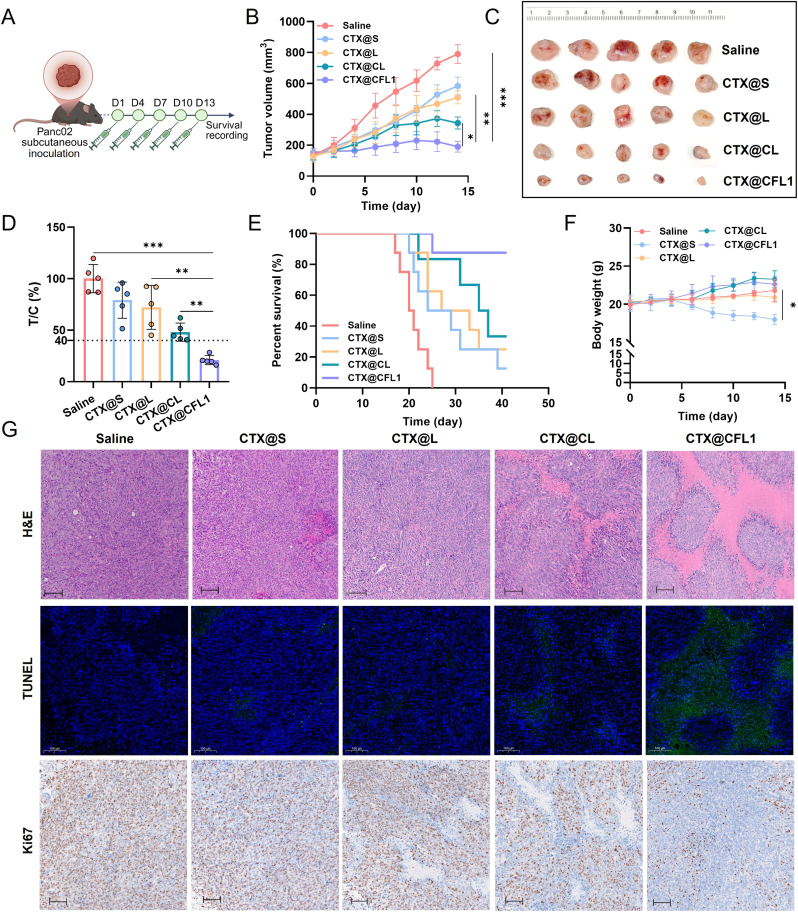
**Improved in vivo antitumor effect of CTX@CFL.** (A) Schematic diagram of experimental scheme for *in vivo* antitumor analysis. (B) Tumor volume curve of Panc02 tumor-bearing mice treated with different liposomes (n = 5). (C) Appearance of harvested tumor tissues from mice at 19th day. (D) Relative tumor proliferation rate (T/C value) of tumor-bearing mice treated with different liposomes (n = 5). (E) Survival curves of tumor-bearing mice treated with different liposomes (n = 8). (F) Change of body weight of treated with different liposomes (n = 5). (G) H&E, TUNEL and Ki67 staining of tumor tissues treated with different liposomes. Scale bar: 100 μm. Results are represented as mean ± SD. ∗p < 0.05, ∗∗p < 0.01, ∗∗∗p < 0.001.

Based on the favorable tumor cellular uptake, enhance penetration, and potent *in vitro* antitumor of CTX@CFL, we proceeded to evaluate its *in vivo* anticancer performance in a subcutaneous Panc02 tumor-bearing mouse mode (Fig. 6A). Over the 15-day treatment period, tumors in the saline group exhibited rapid, aggressive growth, exceeding 800 mm^3^ in volume (Fig. 6B). In contrast, all treatment groups, including free CTX solution (CTX@S), CTX@L, CTX@CL and CTX@CFL1, exhibited varying degrees of tumor growth suppression. Among them, CTX@CFL1 demonstrated the most potent and sustained inhibitory effect, resulting in the smallest tumor volumes throughout the study. Macroscopic observation of excised tumor tissues at the endpoint corroborated this superior antitumor efficacy (Fig. 6C). Quantitative analysis using the relative tumor proliferation rate (T/C value) further distinguished the treatment efficacy (Fig. 6D). The CTX@CFL1 group achieved a T/C value of 21.05%, which is well below the 40% threshold commonly indicative of high antitumor efficacy. This result was notably superior to those of the CTX@CL (48.12%), CTX@L (72.15%), and CTX@S (79.16%) groups, highlighting the critical impact of liposomal flexibility on therapeutic outcomes. Beyond tumor growth inhibition, survival analysis revealed that CTX@CFL1 treatment conferred the longest median survival time (40 days) and the highest survival rate (87.5%). The overall survival benefit ranked as followed: CTX@CFL1 > CTX@CL > CTX@L > CTX@S > saline group (Fig. 6E). Importantly, mice treated with CTX@CFL1 remained stable body weight throughout the treatment period (Fig. 6F), suggesting minimal systemic toxicity and good tolerability of the formulation. In contrast, mice administrated free CTX@S showed obviously body weight loss, likely attributable to the high inherent irritancy and toxicity of the solubilizing excipients [62].

To elucidate the treatment effects at the histopathological level, tumor sections were analyzed by H&E staining, TUNEL apoptosis assay, and Ki67 proliferation immunohistochemistry. As shown in Fig. 6G, tumors from the saline group exhibited densely packed hyperchromatic nuclei, minimal apoptotic cells, and high Ki67 positivity, reflecting a vigorous proliferation and viability. In contrast, all CTX-treated groups showed increased apoptosis and reduced proliferation activity. The most significant effects were observed in the CTX@CFL1 group, which showed extensive apoptotic regions and the lowest Ki67 expression, confirming its superior ability to induce tumor cell death and suppress proliferation. In summary, these *in vivo* results strongly demonstrate that engineering flexibility into cationic liposomes significantly improve the chemotherapeutic efficacy of CTX against solid tumors. This improvement is mechanistically linked to the previously demonstrated enhancements in tumor penetration and cellular uptake, solidifying the potential of flexibility-modulated cationic liposomes as an effective delivery strategy for anticancer drugs.

### 2.7 Biocompatibility and safety of CTX@CFL

**Fig. 7:**
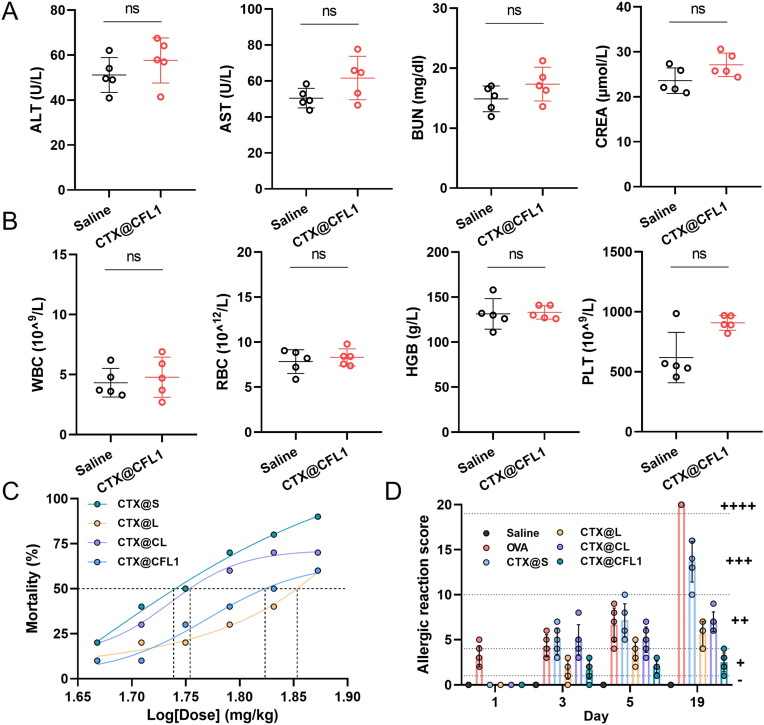
**Biocompatibility and safety of CTX@CFL.** (A) Serum biochemistry and (B) blood routine indicators of mice treated with CTX@CFL1 after 7 days. (C) Acute toxicity of different CTX formulations to health mice (n = 10). (D) Allergic reaction of different CTX formulations to guinea pigs, where the allergic reaction 0, 1∼4, 5∼10, 11∼19 and 20 represented negative (−), weakly positive (+), positive (++), strongly positive (+++), and extremely positive (++++) reaction, respectively (n = 6). Results are represented as mean ± SD. ns means no significant.

Systemic toxicity of nanomedicine is a key index to validate its clinical potential [[63], [64], [65]]. To evaluate the hemocompatibility of CTX@CFL1, a hemolysis assay was conducted across a concentration range of 25 to 100 μg/mL CTX, which covered the dosage used in the antitumor study *in vivo*. The hemolysis rates of CTX@CFL1 were below 5%, indicating negligible hemolytic toxicity (Fig. S12A). Following intravenous injection of CTX@CFL1, no pathological abnormalities were observed in the main organs of mice (Fig. S12B). Furthermore, the serum biochemistry and blood routine test showed no significant abnormality, confirming that CTX@CFL1 did not adversely affect hematological parameters or hepatic and renal functions (Fig. 7A and 7B). The acute toxicity was assessed by determining the median lethal dose (LD_50_) in mice over a 14-day observation period (Fig. 7C). The LD50 of CTX@CFL1 was 66.07 mg/kg, which was higher than that of CTX@CL (56.23 mg/kg) and CTX@S (53.70 mg/kg). These results indicate a favorable acute toxicity profile for the flexible liposomal formulation relative to its counterparts. The allergic reaction was tested in guinea pigs using a standardized scoring system (Table S1). As shown in Fig. 7D, CTX@S elicited a strongly positive reaction similar to the ovalbumin (OVA) positive control, while CTX@CL induced a positive reaction. In contrast, CTX@CFL1 displayed a lower allergic reaction, as indicted by its significantly reduced allergic reaction score. Collectively, these comprehensive safety assessments demonstrate the favorable biocompatibility and safety profile of CTX@CFL1, supporting its potential for further therapeutic development.

## 3 Conclusion

The clinical application of cationic liposomes in solid tumors is fundamentally limited by their propensity for peripheral electrostatic sequestration, which restricts drug access to deeper tumor regions. To overcome this barrier, we investigated flexibility as a key design parameter. We demonstrate that the flexibility of cationic liposomes is a critical determinant governing their tumor delivery efficiency and ultimate therapeutic efficacy. Employing sodium cholate as a membrane-modulating agent, we engineered a cationic flexible liposome (CTX@CFL) with reduced elasticity modulus, enhanced membrane fluidity, and superior deformability. These optimized mechanical properties shifted the primary cellular uptake pathway from endocytosis to membrane fusion, minimized lysosomal entrapment, enhanced transendothelial transport, and promoted deep tumor penetration. Collectively, these mechanisms synergized to achieve superior cytosolic drug delivery, which translated into potent anticancer effects both *in vitro* and *in vivo*. The lead formulation (CTX@CFL1) exhibited an excellent biocompatibility profile and significantly enhanced therapeutic efficacy in a preclinical model, underscoring the clinical potential of flexibility-modulated nanocarriers.

## 4 Materials and methods

### 4.1 Materials

Cabazitaxel, 1,2-dioleoyl-3-trimethylammonium-propane (DOTAP) and 1,6-diphenyl-1,3,5-hexatriene (DPH) were purchased from Sigma-Aldrich (USA). Coumarin 6 (Cou-6), poloxamer 188, poloxamer 407, TPGS, Solutol HS-15 and IR780 were purchased from Aladdin Chemistry Co., Ltd (China). Hoechst 33342 was purchased from KeyGENBioTECH (China). Tween 80 and Span 80 were purchased from J&K Scientific (China). Sodium cholate, sodium deoxycholate, oleic acid, linoleic acid, cholesterol and progesterone, DiO were purchased from Shanghai Yuanye Bio-Technology Co., Ltd (China). Egg yolk lecithin PL-100M was purchased from AVT (Shanghai) Pharmaceutical Tech Co., Ltd (China). RPMI-1640 medium was purchased from Shanghai Life-iLab Biotech Co., Ltd. Chlorpromazine, methyl-β-cyclodextrin and bafilomycin A1 were purchased from KKL Med Inc (USA). Lysosome extraction kit was purchased from Solarbio Science & Technology Co.,Ltd. (China). Cyclin B1 ELISA Kit and cytochrome C ELISA kit were purchased from Abcam Limited (UK). Calcein/PI staining kit, CCK-8 kit and TUNEL cell apoptosis detection kit were purchased from Beyotime Biotechnology Limited (China). Octadecyl Rhodamine B (R18) and tribromoethanol were purchased from Macklin Inc. (China). 2-Methyl-2-butanol was purchased from Energy Chemical (China).

### 4.2 Cell culture and animals

Panc02 cells and HUVECs were cultured in DMEM and ECM with 10% FBS at 37°C in a 5% CO_2_ atmosphere, respectively. C57BL/6 mice were purchased from GemPharmatech Co. Ltd. (China). Animal experiments were performed under protocols approved by the Animal Ethics Committee of Hangzhou Normal University (20241005-03).

### 4.3 CTX@CFL liposome preparation and characterization

2 mg of CTX and 100 mg of egg yolk lecithin PL-100M with 1.2 mol% DOTAP were dissolved in ethanol at 50°C, and then performed rotary evaporation and hydration with 2 mL PBS containing 4 mol% or 8 mol% sodium cholates. The liposome suspension was sonicated at 300 W for 8 min using an Ultrasonic Homogenizer (JY92-IIN, Ningbo Scientz Biotechnology Co., Ltd, China) in an ice bath and dialyzed for 24 h to obtain the finial liposome product. The size distribution and zeta potential of liposomes were evaluated by dynamic light scattering (DLS, 90Plus, Brookhaven, USA). The morphology of liposomes was observed by TEM imaging (Hitachi, Japan). 500 μL of the liposome sample were add to the upper chamber of an ultrafiltration tube (with a molecular weight cut-off of 30 kDa). After centrifugation at 3000 rpm for 30 min, the filtrates were collected and measure the free CTX concentration (C_free_) to calculate the encapsulation efficiency (%) = [1 - (C_free_ × V_free_)/(C_total_ × V_total_)] × 100%. The CTX content in liposomes was determined to calculate the loading rate (%) = (W_CTX_/W_CTX_ + W_carriers_) × 100%.

### 4.4 Drug release *in vitro*

2 mL liposome suspensions were respectively placed into dialysis bags, which were immersed in 200 mL of PBS and stirred at 37°C and 100 rpm. 200 μL samples were taken at 0, 0.5, 1, 2, 4, 8, 12, and 24 h, and the CTX concentration was measured to calculate the cumulative release amount. The release amount at 0.5 h was taken as the burst release amount, and the burst release amounts at 37°C were measured respectively.

### 4.5 Stability test

The liposome samples were stored at 4°C, and the particle size was measured at 0, 2, 4, 6 and 8 days to test the storage stability. The liposome samples were incubated with mice plasma at 37°C in a shaking bath, and the particle size was measured at 0, 4, 8, 12, 24 and 48 h.

### 4.6 Measurement of liposome membrane fluidity

The fluidity of liposomes was analyzed by measuring the fluorescence anisotropy values of 1,6-diphenyl-1,3,5-hexatriene (DPH) labeled liposomes. 1,6-diphenyl-1,3,5-hexatriene (DPH) was dissolved in tetrahydrofuran and added to the liposome suspension (final concentration 2 μM). The mixture was incubated in the dark for 30 min at 37°C with slight shaking to allow DPH to embed in the lipid bilayer. The DPH-labeled liposomes were transferred to a black 96-well plate. The temperature control module of the microplate reader was set up, with temperature intervals of 5°C, starting from 25°C and gradually increasing to 60°C. Each temperature point was equilibrated for 5 min. Fluorescence intensities in the parallel and perpendicular polarization directions, I_1_ and I_2,_ were measured at Ex/Em = 360/430 nm. The fluorescence anisotropy values were calculated by the formula r=(I_1_-I_2_)/(I_1_+2I_2_), and the fluorescence anisotropy-temperature curve was plotted to compare the fluidity of different liposome formulations.

### 4.7 Measurement of liposome elasticity modulus

Langmuir-Blodgett film (KSV NIMA, Biolin Scientific, Sweden) was applied to test the elasticity modulus of liposomes. The typical surface pressure-mean molecular area (π-A) isotherms. The different liposomal formulations were dissolved in chloroform: methanol (v/v, 3:1) at a concentration of 0.5 mM. These solutions were spread on the surface of water phase using a Hamilton syringe (30 μL) and left to equilibrate for 15 min to evaporate the organic solvent. The lipid membrane was compressed by two mobile barriers at a constant rate of 5 mm/min, and the π-A isotherms were obtained by the computer automatically. The compressibility modulus Cs^−1^ (mN/m) = -A×dπ/dA of the lipid membrane was calculated based on the slope (dπ/dA) from the linear region of π-A curve, and then converted to the three-dimensional elastic modulus E (MPa) = Cs^−1^ × (1 - *ν*)/h, where *ν* was the Poisson ratio (0.5) and h was the thickness of the lipid membrane.

### 4.8 Filtration extrusion experiments

1 mL of coumarin 6-labeled liposomes were filtered through 0.2 μm nylon syringe filters, and the filtrate was observed. The fluorescence value of filtrate and unfiltered liposomes was measured at Ex/Em = 450/505 nm by a microplate reader (Infinite 200 PRO, Switzerland), and fluorescence percentage of filtrate was calculated.

### 4.9 Ultracentrifugation experiments

Coumarin 6-labeled liposomes were loaded into the upper compartment of the 0.2 μm Nanosep ultracentrifuge tubes (Cytiva, USA) and centrifuged to obtain the filtrate. The samples were determined for fluorescence at Ex/Em = 450/505 nm by a microplate reader, derived count rates and particle size by Zetasizer Nano ZS90 (Malvern, UK). The uncentrifuged sample was used as a reference to calculate the fluorescence percentage (%) and count percentage (%).

### 4.10 Membrane fusion assay

Octadecyl Rhodamine B (R18) was used as a fluorescent molecular probe. Liposomes were co-incubated with R18 (Macklin, China) for 30 min at room temperature, which was centrifugated and resuspended in PBS. Panc02 cells were seeded in 96-well plates and incubated overnight, after which 150 μL of R18-labeled liposome suspension were added. 100 μg/mL of Z-Phe-Phe-Phe-OH (membrane fusion inhibitor) was also preadded and performed the test as a control. The fluorescence change (Ex/Em = 560/590 nm) was detected using a microplate reader for 30 min with an interval of 60 s.

Liposomes incorporated with NBD-PE and Rho-DHPE (1:1, W/W) were prepared to detect the fluorescence resonance energy transfer (FRET). The fluorescence intensity of NBD was measured by a microplate reader to calculate the increased NBD fluorescence (%)= (NEB-NEB_min_)/(NEB_max_-NEB_min_). The NBD_max_ was the final NBD fluorescence when added 1% Triton X-100 to solubilize the liposomes.

To further confirm the membrane fusion pathway, the direct detection of mixing effect the aqueous cavity of liposomes with the cell cytoplasm was performed. The calcein-loaded CTX@CFL formulations were prepared by hydration in PBS with calcein (120 mM) and NaOH (1 M), and dialyzed (MW: 3000 Da) in PBS at 4°C for 24 h. Then, the Panc02 cells were incubated with calcein-loaded CTX@CFL at 37°C for different time, and the treated cells were collected to detect fluorescence value at Ex/Em of 489/515 nm.

### 4.11 Measurement of cellular uptake

2 × 10^5^ cells of Panc02 suspension were seeded in 20-mm glass-bottom cell culture dishes (NEST Biotechnology Co. Ltd. Wuxi, China) and incubated with coumarin 6-labeled liposomes for 1 h at 37°C. Then, the Panc02 cells were stained with Hoechst 33342 to be detected by confocal laser scanning microscope (Olympus, Japan). The fluorescence intensity of the collected Panc02 cells was tested by flow cytometry (BD, FACSCalibur). To investigate the uptake pathway, Panc02 cells were pre-incubated with endocytosis inhibitors CPZ (20 μM), HP-β-CD (10 mM), amiloride (40 μM), 0.01% w/v sodium azide or Z-Phe-Phe-Phe-OH (100 μg/mL) at 37°C for 30 min, and further incubated with liposomes for flow cytometry analysis. After incubation with coumarin 6-labeled liposomes, Panc02 cells or 50 nM Bafilomycin A1 preincubated cells were treated with lysosome extraction kit (Solarbio, China) to obtain lysosome suspension to test the fluorescence intensity by a microplate reader at Ex/Em = 484/501 nm.

### 4.12 Transendothelial assay by transwell insert system

HUVEC in serum-free medium were seeded into transwell insert (Corning, USA) in a 24-well plate. Then, 50 ng/mL TNF-α were added to stimulate HUVEC for 24 h. The transepithelial electric resistance (TEER) was tested by Millicell ERS-2 (Millipore, USA). Coumarin 6-labeled liposomes were added into the upper chamber, and incubated for 2 h at 37°C. The sample was collected from the lower chamber to test the fluorescence intensity and particle size.

### 4.13 Permeability assay of tumor spheres

5 × 10^3^ cells of Panc02 suspension was added to a 96-well ultra-low adhesion plate, and centrifuge at 1000 rpm for 2 min at room temperature. Then, the plate was placed in the cell incubator and kept it tilted at an angle of 30° for 7 d. When the size of tumor spheres reached 400 μm, the spheres were transferred to a confocal culture dish and incubated with 100 μL of coumarin 6-labeled liposomes for 2 h at 37°C. They were washed with PBS, fixed with 4% paraformaldehyde and observe under a confocal microscope for Z-axis 3D imaging, with a scanning step size of 50 μm. The mean fluorescence intensity of spheres in central and peripheral zone was quantified by Image J software. The tumor spheroid permeability rate was calculated by the percentage of fluorescence intensity in central and total zone.

### 4.14 Cytotoxicity measurement

1 × 10^4^ cells of Panc02 suspension were seeded in a 96-well plate and incubated overnight. Different liposomes at the CTX concentration of 0.1 to 1000 ng/mL and the carries without CTX were added and incubated for 24 h. The cell viability was determined by a CCK-8 kit.

### 4.15 Detection of cyclin B1 and cytochrome C

Panc02 cells were incubated with liposomes for 48 h at 37°C and obtained the lysate. The expression level of cyclin B1 was detected using a cyclin B1 ELISA Kit (Abcam, UK) according to the manufacturer's instructions. The released cytochrome C in lysate was tested by a cytochrome C ELISA kit (Abcam, UK) according to the manufacturer's instructions.

### 4.16 Antitumor effect *in vitro* by transwell insert

HUVEC in serum-free medium were seeded into transwell insert (Corning, USA) in a 24-well plate, and Panc02 cells were seeded into the lower chamber. 50 ng/mL TNF-α were added to stimulate HUVEC for 24 h. Different CTX liposomes at 10 μg/mL were added into the upper chamber, and incubated for 2 h at 37°C. Then, the upper chamber was washed with PBS, and cultured for 24 h. The Panc02 cells in the lower chamber was stained by Calcein/PI staining kit (Beyotime, China) to analyze the live or dead cells. The cell viability of Panc02 was tested by CCK-8 kit.

### 4.17 Apoptosis effect *in vitro* in tumor spheres

5 × 10^3^ cells of Panc02 suspension was added to a 96-well ultra-low adhesion plate, and centrifuge at 1000 rpm for 2 min at room temperature. Then, the plate was placed in the cell incubator and kept it tilted at an angle of 30° for 7 d. When the size of tumor spheres reached 400 μm, the spheres were transferred to a confocal culture dish and incubated with 10 μg/mL liposomes for 2 h. They were washed with PBS, fixed with 4% paraformaldehyde and performed apotosis staining by a TUNEL cell apoptosis detection kit (Beyotime, China) according to the manufacturer's instructions to observe under a confocal microscope. The mean fluorescence intensity of spheres in central and peripheral zone was quantified by Image J software.

### 4.18 Tumor accumulation *in vivo*

Subcutaneous Panc02 tumor-bearing mouse model was used to evaluate the tumor accumulation of liposomes. In brief, 5 × 10^5^ of Panc02 cells were subcutaneously injected into the right groin of female C57BL/6 mice. When the tumor volume reached around 500 mm^3^, they were randomly separated into different groups. 100 μL of free IR780 and IR780-labeled liposomes were intravenously injected into the mice. The fluorescence images were acquired with IVIS Spectrum (PerkinElmer, USA) at 0.5, 2, 4, 8, 12, 24, 48 and 72 h. At last, the mice were sacrificed to harvest the major organs and perform fluorescence imaging. The tumor tissues were sliced and stained with DAPI to observe the distribution of liposome in tumor by fluorescence microscopy and the mean fluorescence intensity was measured by Image J software.

### 4.19 Antitumor efficacy *in vivo*

Panc02 cells (5 × 10^5^) were subcutaneously injected into the right groin of mice. After tumor inoculation for 7 days, C57BL/6 tumor-bearing mice were randomly divided into 5 groups: Saline, CTX@S, CTX@L, CTX@CL and CTX@CFL1. Liposomal formulations (2 mg/kg of CTX) were intravenously injected into tumor-bearing mice at day 1, 4, 7, 10 and 13. The tumor volume (V_tumor_ = a×b^2^/2, a: long diameter, b: short diameter, mm) and body weight were calculated every 2 days, and the survival period was monitored throughout the study. The relative tumor proliferation rate was calculated by T/C = T_RTV_/C_RTV_×100% (T_RTV_: RTV value of treated group, C_RTV_: RTV value of control group, RTV=Vt/V_0_, V_0_: initial tumor volume, Vt: tumor volume at t day). After 19 days, tumor-bearing mice were sacrificed to collect tumor for H&E (H&E staining kit, OriLeaf, Shanghai, China), TUNEL staining (Cat 40306 ES, Yeasen, Shanghai, China) and Ki67 analysis (Ki-67 Recombinant Rabbit mAb, bsm-52455R, Bioss, USA)

### 4.20 Biocompatibility and safety analysis

The hemolysis assay of CTX@CFL1 was tested from 25 μg/mL to 100 μg/mL of CTX. CTX@CFL1 liposomes were incubated with 2% red blood cell suspension for 2 h at 37°C. After centrifugation, the supernatant was determined at 540 nm to calculate hemolysis ratio. Saline and pure water were used at negative and positive control. The mice blood was collected after treatment with CTX@CFL1 to perform the blood routine examination and the serum biochemical indicators test. C57BL/6 mice treated with saline or CTX@CFL1 were executed on the 14th day and the main tissues were collected for histological examination by H&E staining. To perform the acute toxicity test, the healthy mice with a body weight of 22-25 g were randomly divided into four groups and intravenous injected with CTX@S, CTX@L, CTX@CL and CTX@CFL1 respectively, with the CTX dose of 46.65, 51.16, 56.21, 61.76, 67.85 and 74.55 mg/kg. The death of mice within 14 days was recorded and the LD50 was calculated. The allergic reaction of liposomes was tested in guinea pigs. Healthy guinea pigs with a body weight of 300-400 g were randomly divided into six groups. They were sensitized by intravenous injection of 0.5 mL saline, OVA (5 mg/mL), CTX@S, CTX@L, CTX@CL and CTX@CFL1 respectively, once every other day for a total of 3 times. 14 days after the last administration, the guinea pigs were challenged with twice dose of the corresponding drug. Within 30 min after administration, the allergic reaction symptoms of the guinea pigs were observed and scored, and the longest observation time was 3 h. The allergic reaction score of 0, 1∼4, 5∼10, 11∼19 and 20 represented negative (−), weakly positive (+), positive (++), strongly positive (+++), and extremely positive (++++) reaction, respectively.

### 4.21 Statistical analysis

All statistical analyses were performed using GraphPad Prism software. Results are represented as mean ± standard deviation (SD). A two-tailed Student's t-test was used to determine the significance between the two groups, and comparisons of multiple groups were performed using one-way ANOVA. ns means no significant. The level of statistical significance was defined as ∗p < 0.05, ∗∗p < 0.01, ∗∗∗p < 0.001.

## CRediT authorship contribution statement

**Lifeng Luo:** Conceptualization, Funding acquisition, Investigation, Methodology, Writing – original draft. **Xiaonan Liu:** Methodology, Validation, Visualization. **Yuqing Cai:** Methodology, Validation, Visualization. **Yiran Zhang:** Formal analysis, Project administration, Validation. **Yifan Zhao:** Investigation, Validation. **Zhenqiang Song:** Formal analysis, Visualization. **Ziwen Meng:** Formal analysis, Visualization. **Tian Xie:** Conceptualization, Funding acquisition, Resources, Writing – review & editing. **Fenghua Meng:** Supervision, Writing – review & editing. **Wenxing Gu:** Conceptualization, Supervision, Writing – review & editing.

## Declaration of competing interest

The authors declare that they have no known competing financial interests or personal relationships that could have appeared to influence the work reported in this paper.

## Data Availability

Data will be made available on request.
