# Supplementary material for: A flexibility-driven delivery strategy for cationic liposomes to enhance tumor penetration and promote membrane fusion-mediated cellular entry
Source: Mater Today Bio. 2026 Jun 18;39:103370. doi: 10.1016/j.mtbio.2026.103370 (PMC13316053; doi:10.1016/j.mtbio.2026.103370)
Supplement: Multimedia component 1 [file mmc1.docx]

Supplementary Materials

**A Flexibility-Driven Delivery Strategy for Cationic Liposomes to Enhance Tumor Penetration and Promote Membrane Fusion-Mediated Cellular Entry**

Lifeng Luo ^a^, Xiaonan Liu ^a^, Yuqing Cai ^a^, Yiran Zhang ^a^, Yifan Zhao ^a^, Zhenqiang Song ^a^, Ziwen Meng ^a^, Tian Xie ^a,^*, Fenghua Meng ^b,^*, Wenxing Gu ^a,^*

^a^ School of Pharmacy, Zhejiang Provincial Key Laboratory of Anti-Cancer Chinese Medicines and Natural Medicines, Engineering Laboratory of Development and Application of Traditional Chinese Medicines, Collaborative Innovation Center of Traditional Chinese Medicines of Zhejiang Province, Hangzhou Normal University, Hangzhou 311121, PR China.

^b^ Biomedical Polymers Laboratory, College of Chemistry, Chemical Engineering and Materials Science, Soochow University, Suzhou 215123, PR China

* Corresponding authors: gu.wenxing@hznu.edu.cn (W. Gu), fhmeng@suda.edu.cn (F. Meng), tianxie@hznu.edu.cn (T. Xie)

**Supplementary figures:**


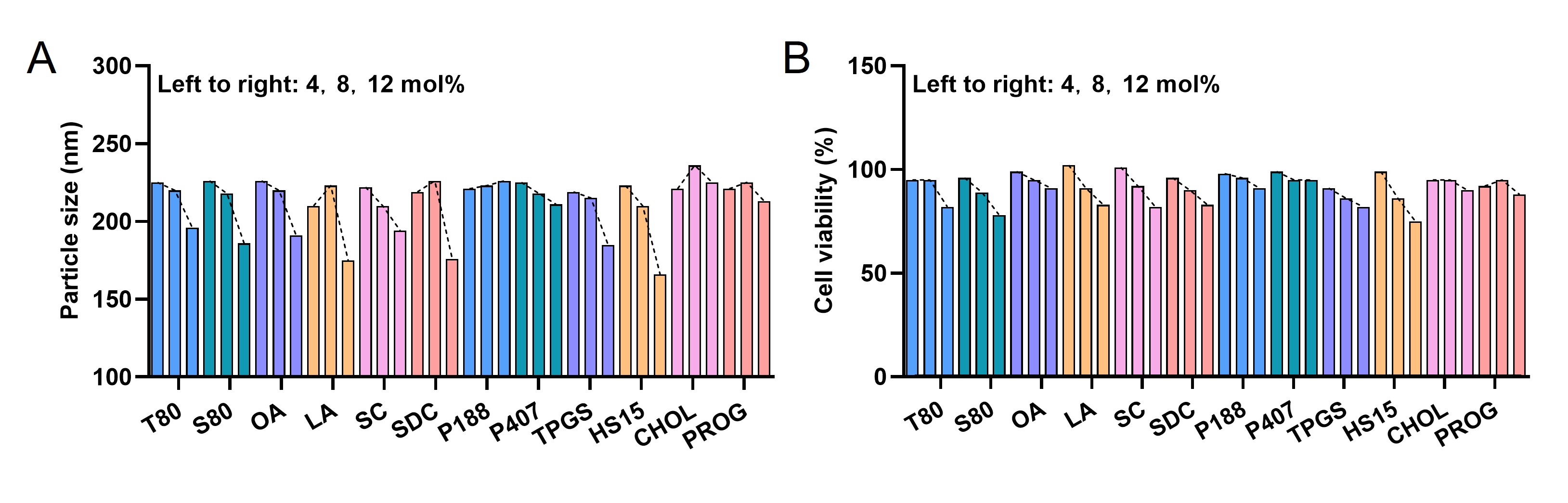


**Fig. S1.** **Influence of different flexibilizers on the particle size and cytotoxicity of cationic liposomes.** (A) Particle size of different cationic liposomes. (B) Cell viability of Panc02 tumor cells incubated with different cationic liposomes.


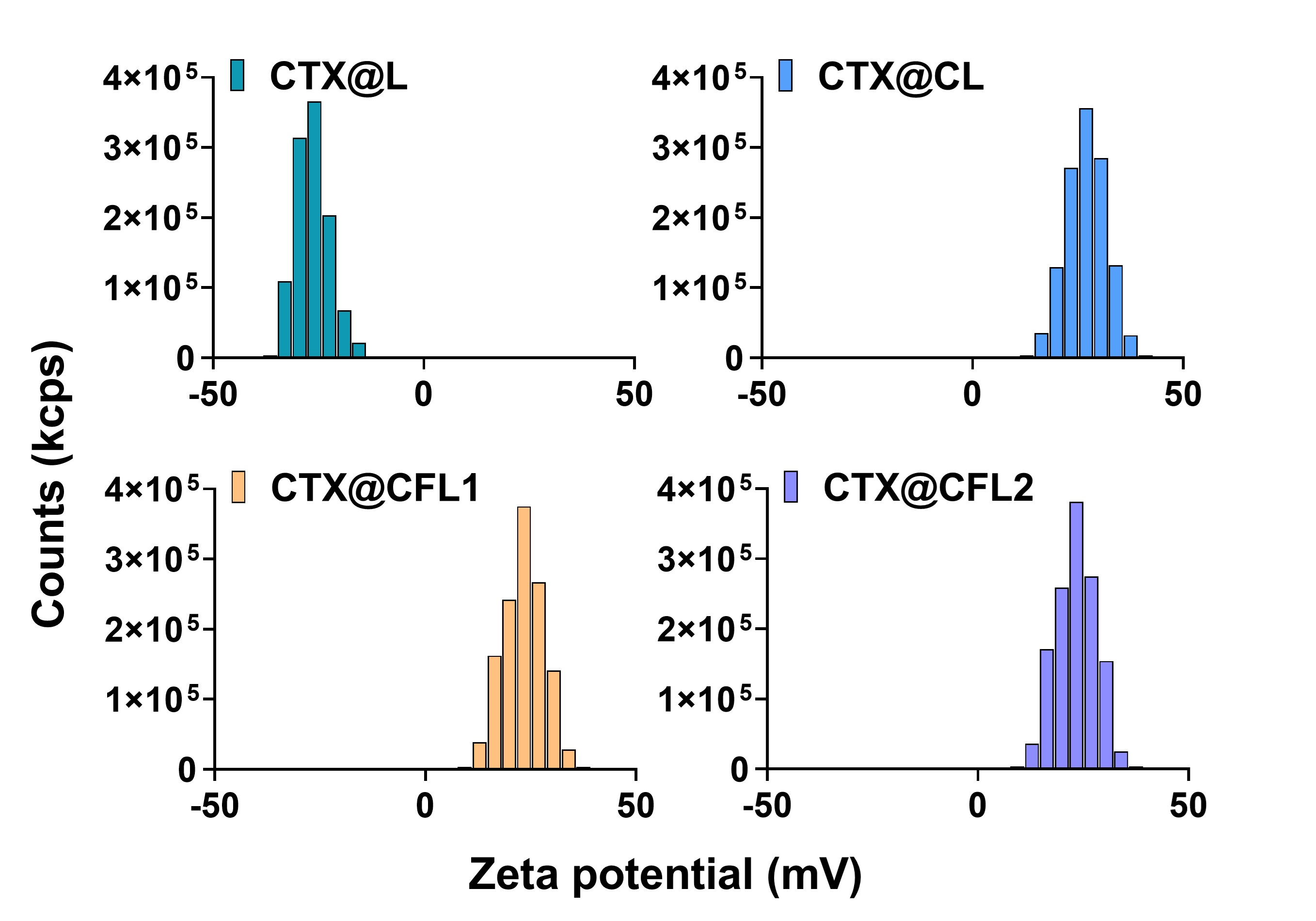


**Fig. S2.** **Zeta potential of different liposomes.**


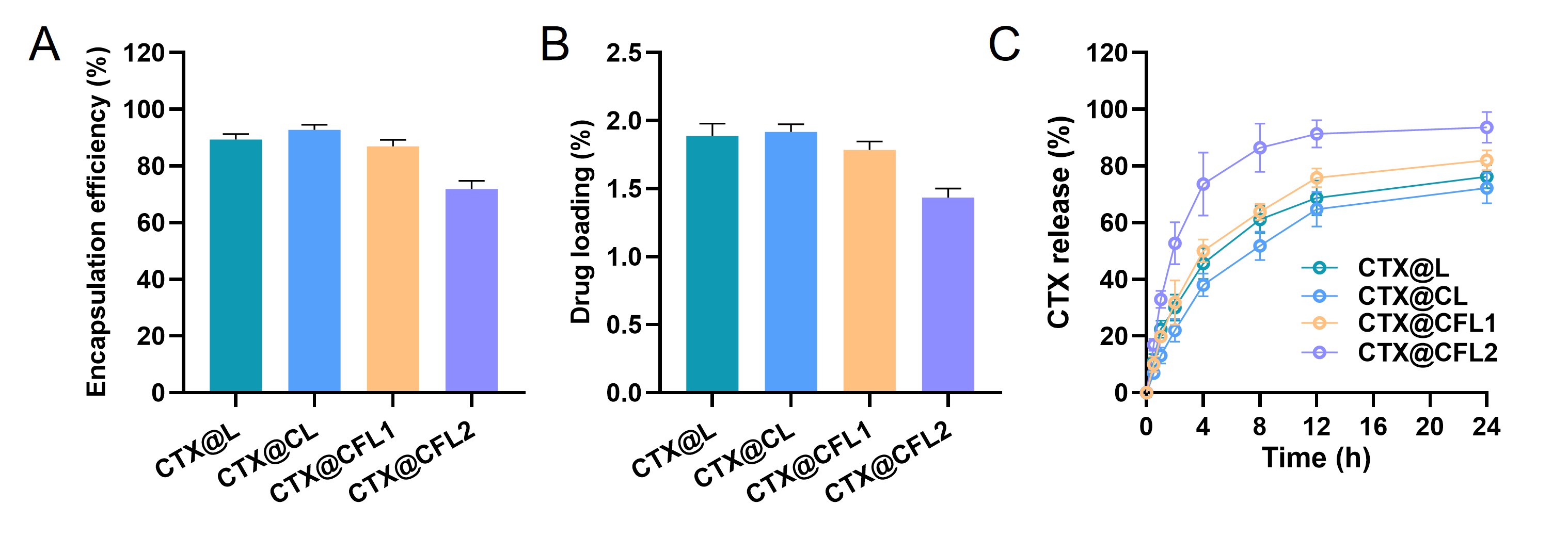


**Fig. S3. Characterization of drug loading in different liposomes.** (A) Encapsulation efficiency of different liposomes (n=3). (B) Drug loading rate of different liposomes (n=3). (C) Drug release curves of different liposomes (n=3). Results are represented as mean ± SD.


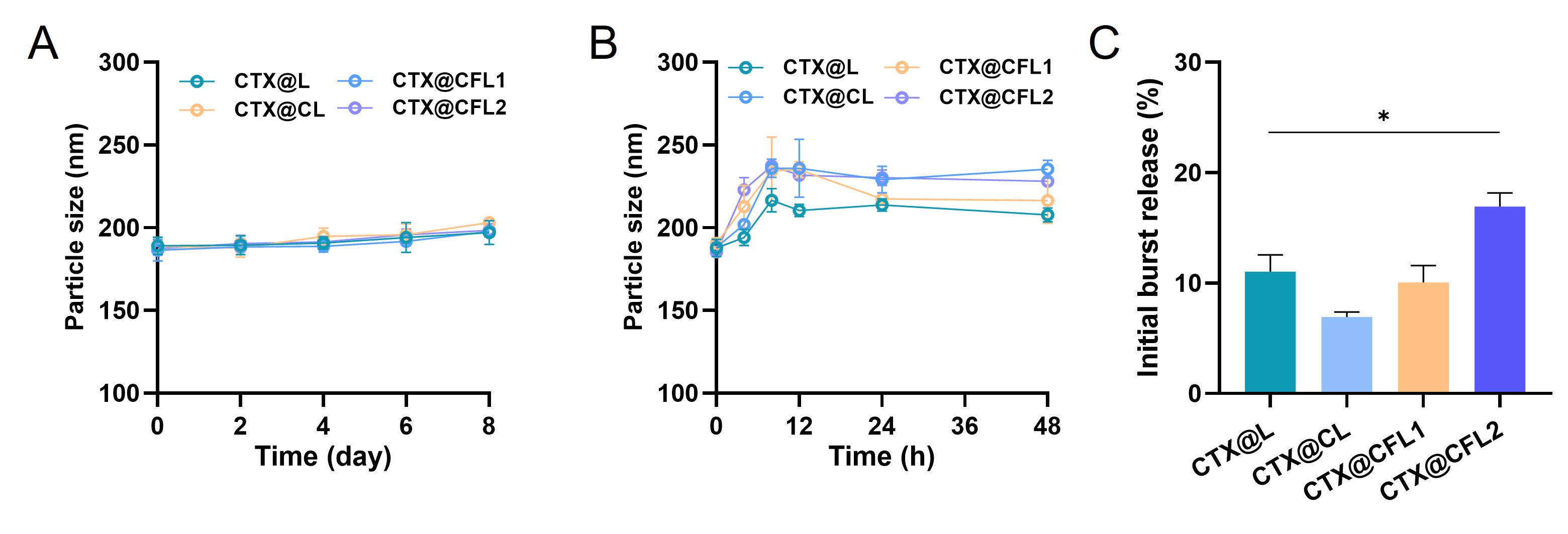


**Fig. S4. Physical stability of different liposomes.** (A) Storage stability of liposomes at 4℃ for 8 days (n=3). (B) Plasma stability of liposomes at 37℃ for 48 h (n=3). (3) Burst release rate of liposomes at 37℃ (n=3). Results are represented as mean ± SD. *p < 0.05.


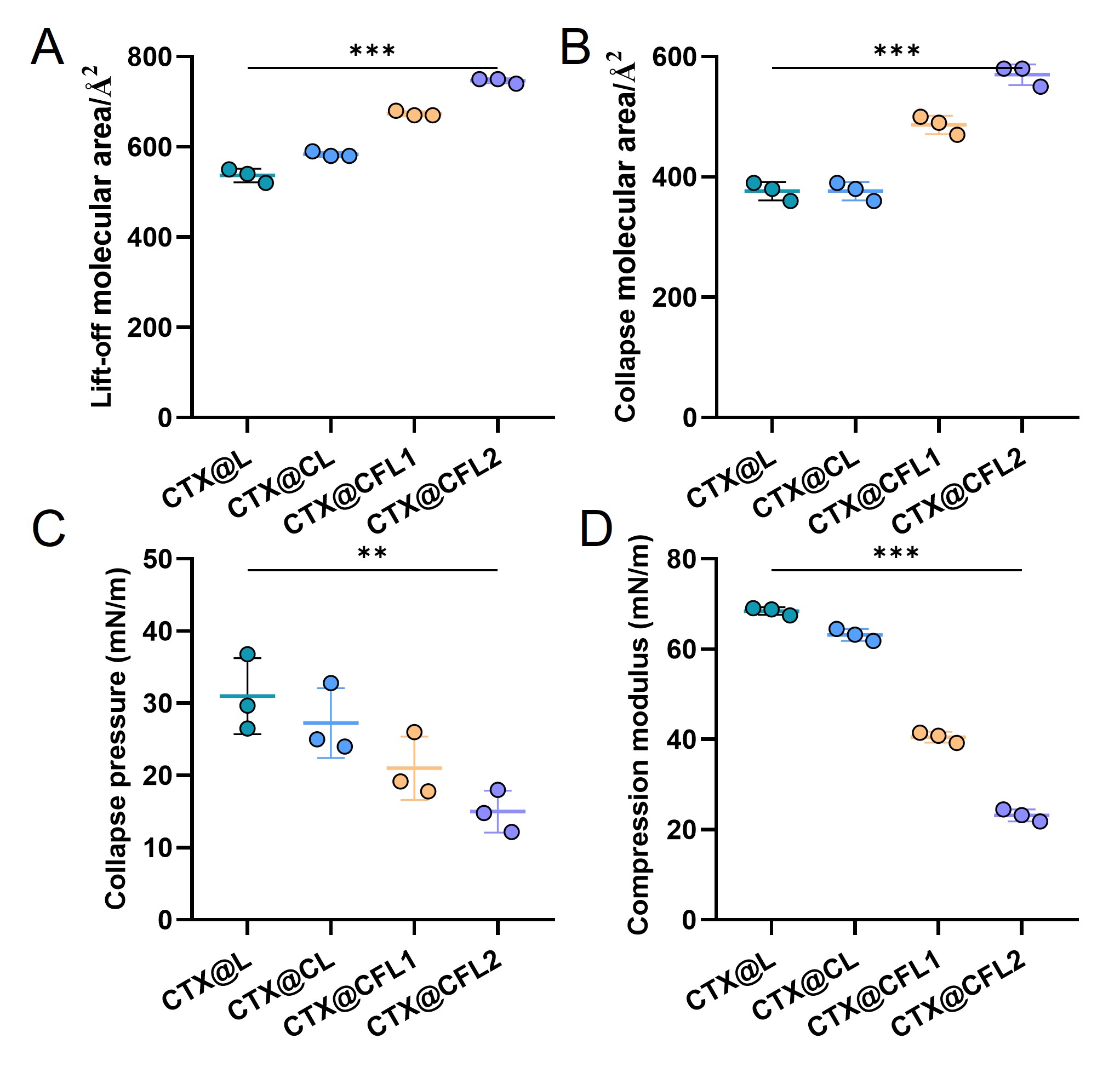


**Fig. S5. π-A curve-derived liposome membrane properties of CTX@CFL.** (A) Lift-off molecular area, (B) collapse molecular area and (C) collapse pressure of π-A curve of different liposomes (n=3). (B) Compression modulus of different liposomes (n=3). Results are represented as mean ± SD. **p < 0.01, ***p < 0.001.


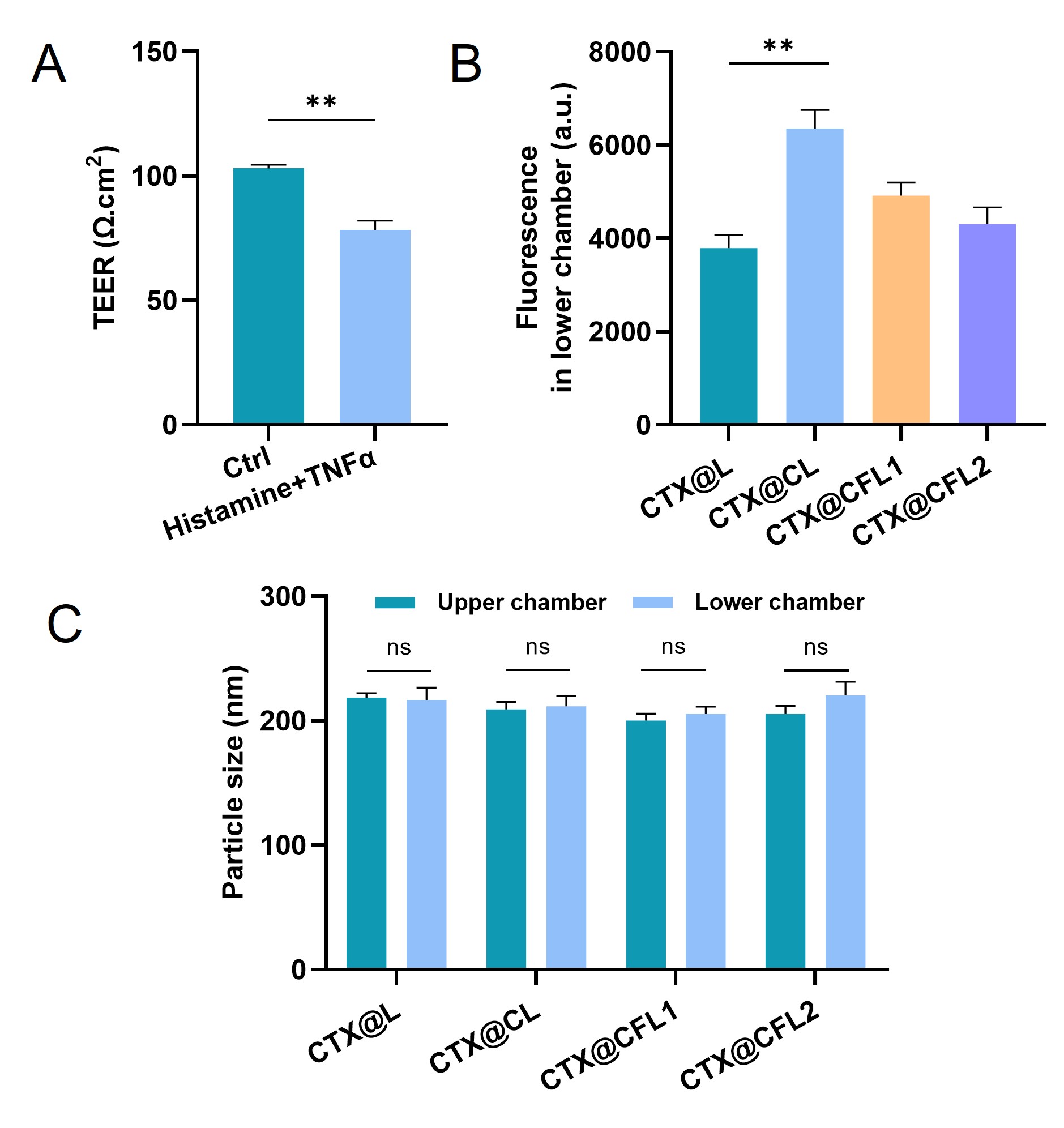


**Fig. S6. Transendothelial analysis of liposomes in a transwell insert system.** (A) TEER value of endothelial layer on the transwell insert when pretreated with 10 μM histamine and 50 ng/mL TNF-α for 4 h (n=3). (B) Fluorescence intensity in lower chamber of a normal transwell system after addition of coumarin 6-labeled liposomes in the upper chamber for 2 h at 37℃ (n=3). (C) Particle size of sample from the upper and lower chamber of the transwell system after different liposomes were added into the upper chamber for 2 h at 37℃ (n=3). Results are represented as mean ± SD. ns means no significant. **p < 0.01.


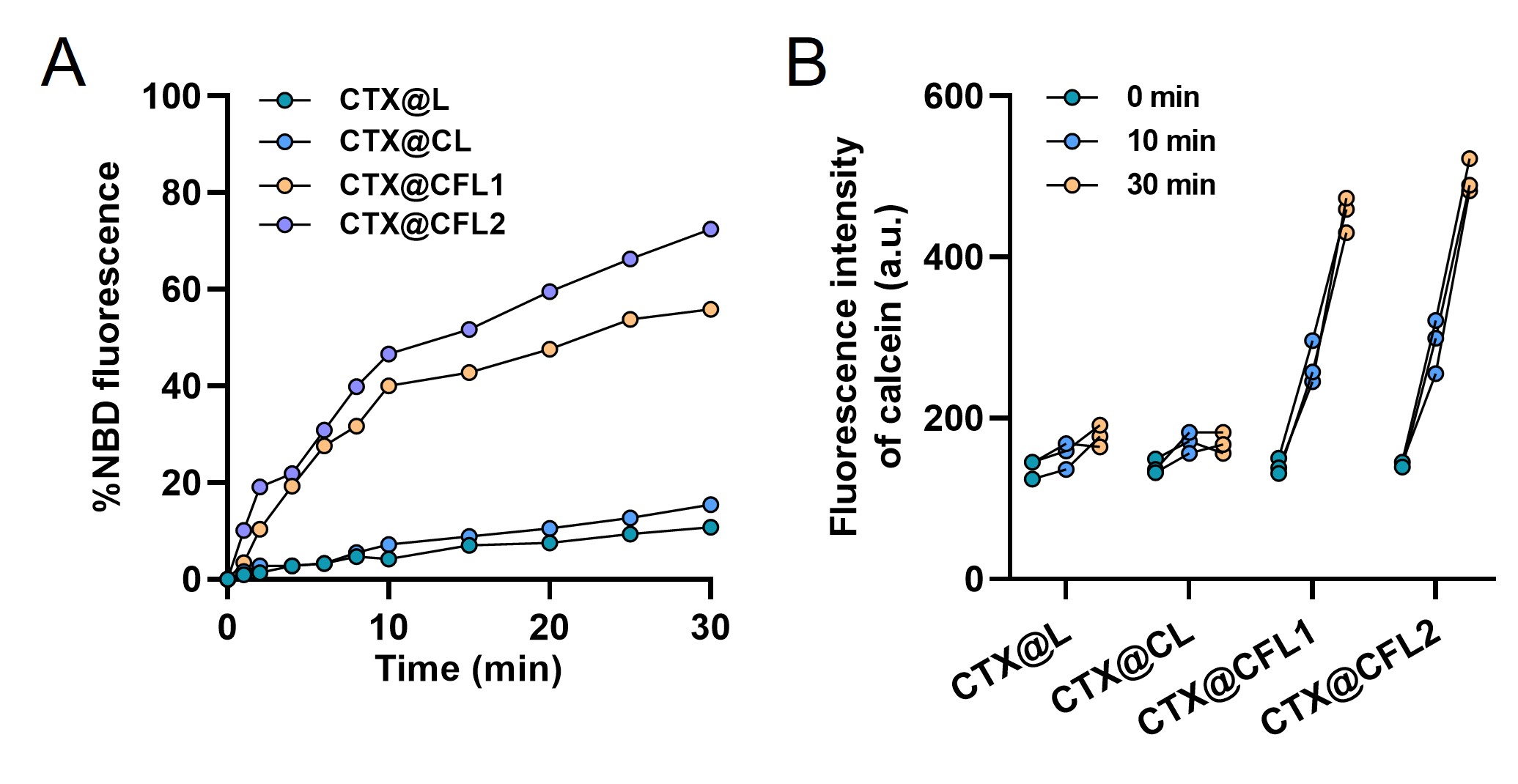


**Fig. S7. Membrane fusion analysis.** (A) NBD fluorescence percentage of cells treated with different liposomes for different time in FRET test. (B) Fluorescence intensity of calcein in the mix system of liposomes with Panc02 cells for different time (n=3).


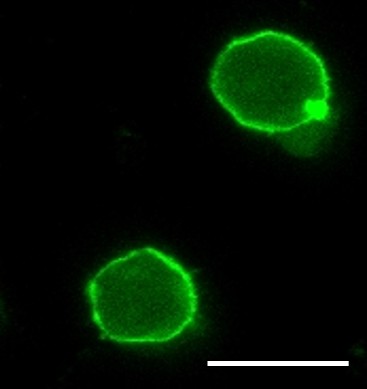


**Fig. S8. Cell membrane integrity analysis.** Fluorescence image of DiO-labeled tumor cells when incubated with CTX@CFL1 for 2 h at 37℃. Scale bar: 20 μm.


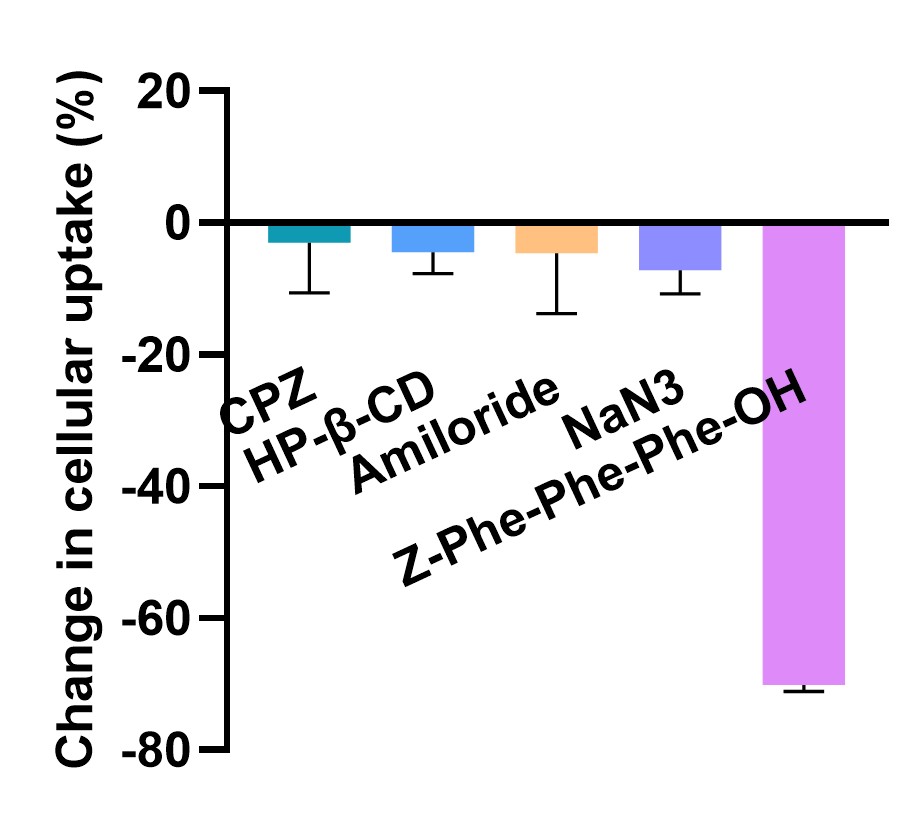


**Fig. S9. Cellular uptake analysis after treatment of different inhibitors.** Change of cellular uptake level of tumor cells pre-incubated with inhibitors chlorpromazine (CD), methyl-β-cyclodextrin (HP-β-CD), macropinocytosis inhibitor amiloride and membrane-fusion restrainer Z-Phe-Phe-Phe-OH (n=3). Results are represented as mean ± SD.


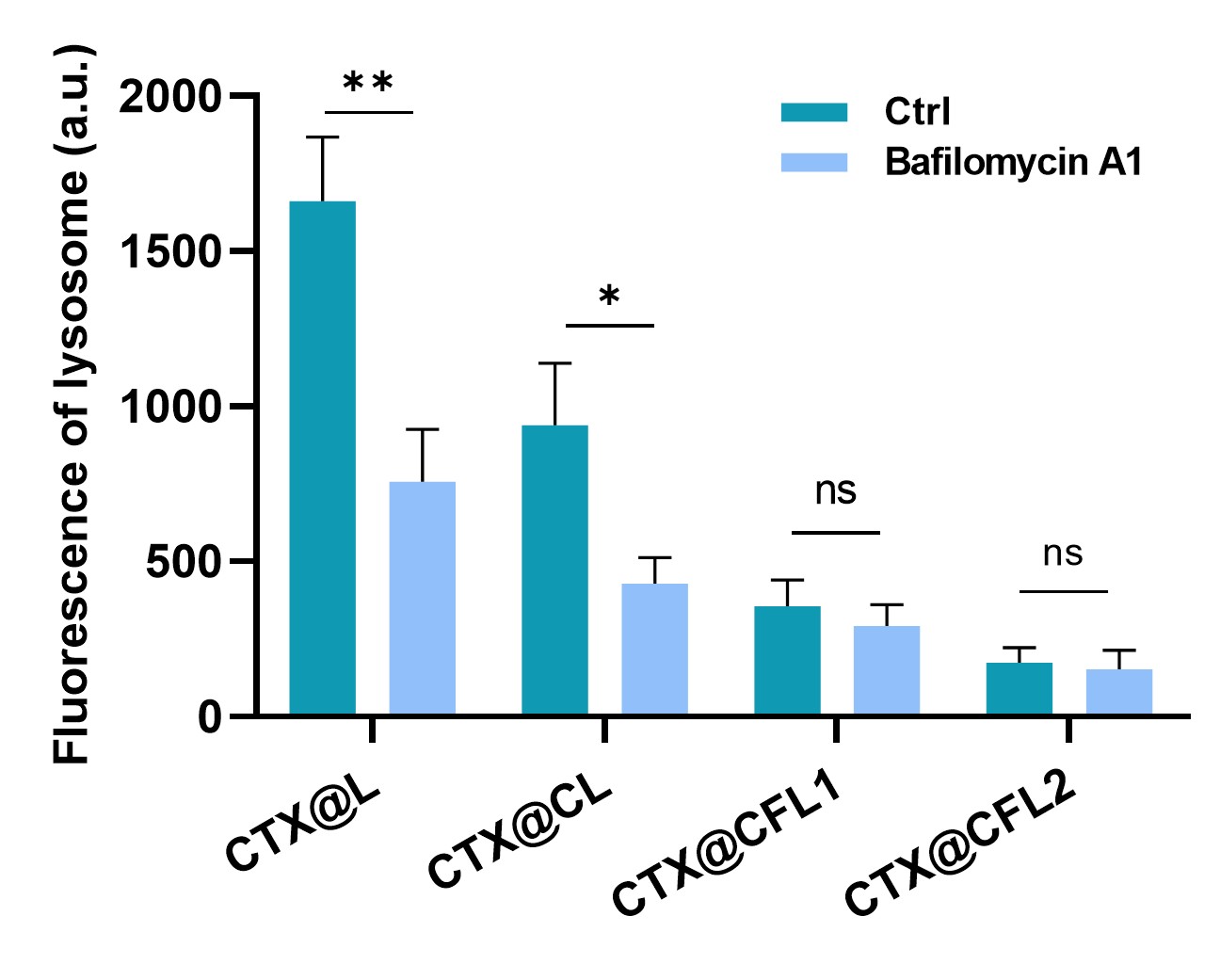


**Fig. S10. Lysosomal accumulation analysis of CTX@CFL.** Quantified fluorescence intensity of extracted lysosome from tumor cells incubated with DiO-labeled liposomes at 37℃ for 60 min (n=3). Results are represented as mean ± SD. ns means no significant. *p < 0.05, **p < 0.01.


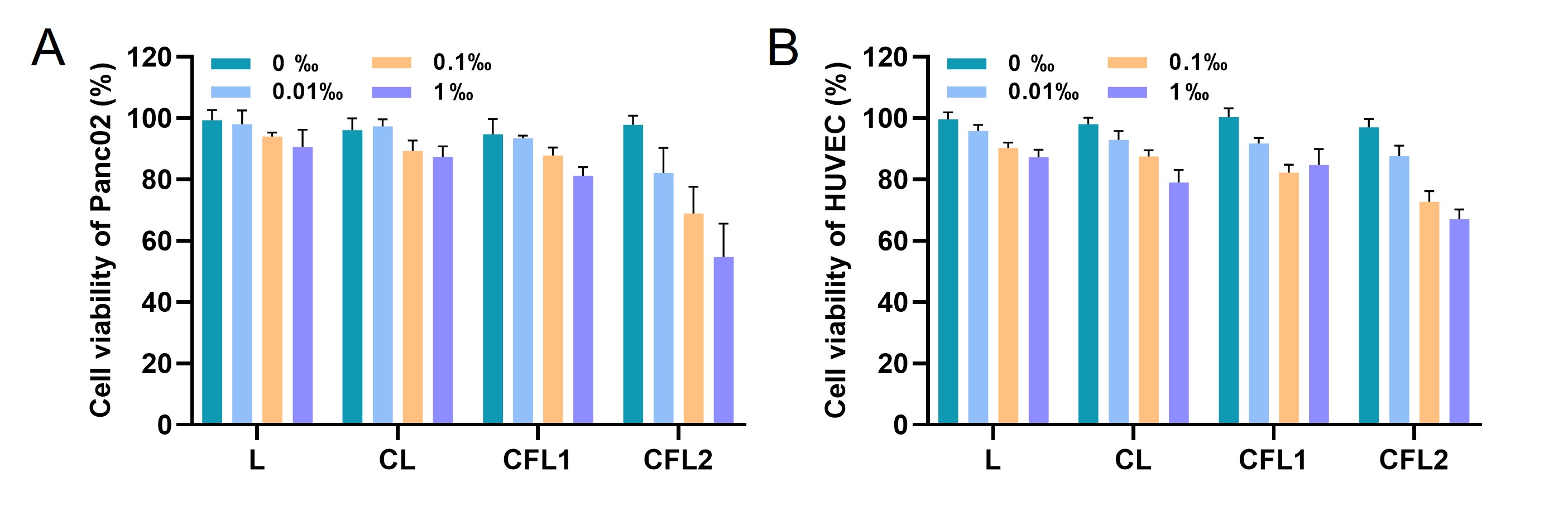


**Fig. S11. Cytotoxicity assay of liposomal vehicle.** Cell viability of (A) Panc02 tumor cells and (B) HUVEC when incubated with different liposomal vehicles at dilution rate of 0‰, 0.01‰, 0.1‰ and 1‰ at 37℃ for 24 h (n=6). Results are represented as mean ± SD.


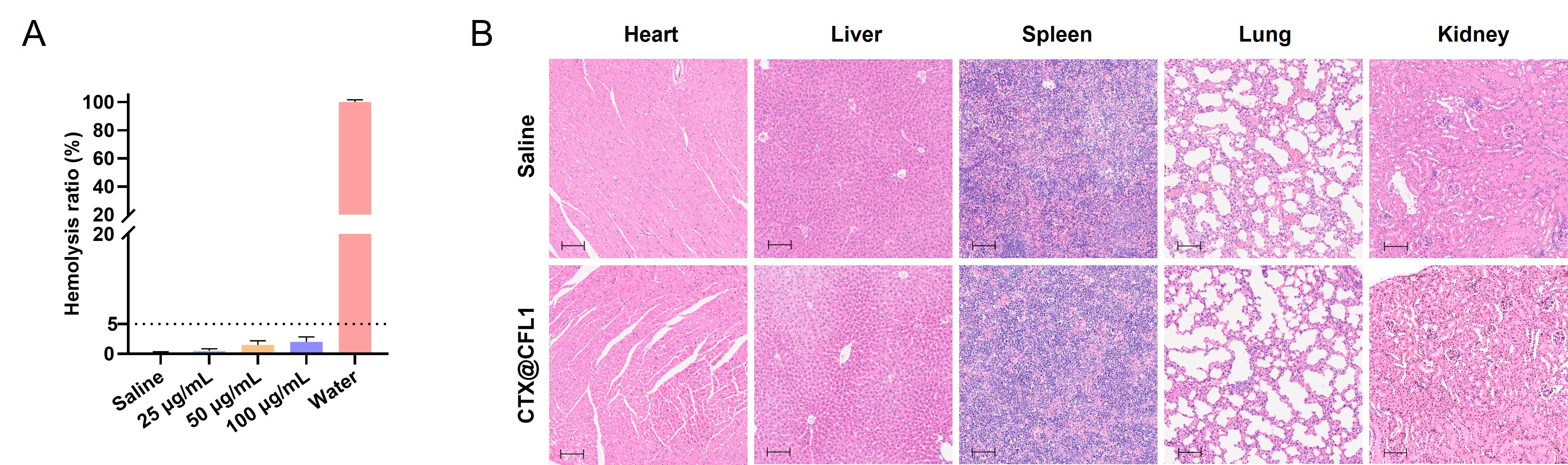


**Fig. S12. Biocompatibility of CTX@CFL.** (A) Hemolysis ratio of CTX@CFL1 *in vitro* (n=3). (B) H&E staining of the main organs of mice treated with CTX@CFL1 after 14 days. Scale bar: 100 μm.

**Table.S1 Allergic reaction score**

| Score | Symptom | Score | Symptom | Score | Symptom |
| --- | --- | --- | --- | --- | --- |
| 0 | Normal | 7 | Polypnea | 14 | Instability of gait |
| 1 | Dysphoria | 8 | Urination | 15 | Jump |
| 2 | Piloerection | 9 | Defecation | 16 | Gasp |
| 3 | Tremble | 10 | Lacrimation | 17 | Spasm |
| 4 | Scratching the nose | 11 | Dyspnea | 18 | Spin |
| 5 | Sneeze | 12 | Wheezing rale | 19 | Tidal breathing |
| 6 | Cough | 13 | Purpura | 20 | Death |
